# Neural Basis for Economic Saving Strategies in Human Amygdala-Prefrontal Reward Circuits

**DOI:** 10.1016/j.cub.2016.09.016

**Published:** 2016-11-21

**Authors:** Leopold Zangemeister, Fabian Grabenhorst, Wolfram Schultz

**Affiliations:** 1 Department of Physiology, Development and Neuroscience, University of Cambridge, Downing Street, Cambridge, CB2 3DY, UK

## Abstract

Economic saving is an elaborate behavior in which the goal of a reward in the future directs planning and decision-making in the present. Here, we measured neural activity while subjects formed simple economic saving strategies to accumulate rewards and then executed their strategies through choice sequences of self-defined lengths. Before the initiation of a choice sequence, prospective activations in the amygdala predicted subjects’ internal saving plans and their value up to two minutes before a saving goal was achieved. The valuation component of this planning activity persisted during execution of the saving strategy and predicted subjects’ economic behavior across different tasks and testing days. Functionally coupled amygdala and prefrontal cortex activities encoded distinct planning components that signaled the transition from saving strategy formation to execution and reflected individual differences in saving behavior. Our findings identify candidate neural mechanisms for economic saving in amygdala and prefrontal cortex and suggest a novel planning function for the human amygdala in directing strategic behavior toward self-determined future rewards.

## Highlights

• Neural activity was measured during planning and execution of economic saving
• Amygdala activity predicted the planned number of saving steps and their value
• Amygdala-frontal circuits coded plan components and individual saving differences
• The results suggest a new function for the human amygdala in economic planning

## Teaser

Economic saving is critical for the welfare of individuals and societies. Zangemeister et al. show prospective activities in human amygdala and prefrontal cortex that reflect economic saving plans and individual differences in saving behavior.

## Introduction

Economic saving is an elaborate form of planned behavior characterized by dynamic, sequential choices and a focus on self-defined future reward [1, 2]. Successful saving is a key determinant of the welfare of individuals and societies, which impacts entire economic systems [3]. Theories in psychology, economics, and reinforcement learning have identified basic principles that underlie planned behaviors involving rewards, such as economic saving: a two-stage process that distinguishes the initial formation of a behavioral strategy from its subsequent execution [1, 4], and a valuation component that directs behavioral strategies toward future rewards [5]. Here, we used fMRI to measure neural activity in an economic reward-saving paradigm that modeled these principles by separating the formation of a reward-based strategy from its execution through sequential choices.

Based on human lesion [6] and neuroimaging evidence and single-cell recordings in monkeys [7], cognitive and action planning are traditionally associated with the frontal lobes. Other prospective functions, such as episodic future thinking and spatial navigation, are associated with medial temporal lobe structures [8, 9]. However, much less is known about how the brain mediates the influence of rewards on planning, despite their crucial importance in directing strategy formation and execution [1, 4, 5]. Studies using intertemporal choice paradigms have uncovered human brain systems for the subjective valuation of delayed rewards [10, 11, 12]. More recent investigations of complex multistep reinforcement learning showed that frontal-striatal systems evaluate reward outcomes associated with externally defined choice paths [13, 14]. These studies identified critical neural components for prospective reward valuation but did not address the key features of planned economic saving, which involve the internal construction of a reward-directed strategy and its subsequent execution through choice sequences of self-defined length [1, 3].

Based on recent single-neuron evidence in non-human primates [15, 16], we hypothesized that in the current study the human amygdala would show prospective activity related to subjects’ economic saving strategies. Our hypothesis was further motivated by evidence of amygdala functions in basic reward valuation [17, 18, 19, 20, 21, 22], processing of economic choice variables [23, 24], and decision-making [25, 26, 27]. We also expected the involvement of prefrontal cortex areas, based on their known valuation, cognitive control, and decision functions [11, 28, 29, 30, 31, 32].

We designed a sequential economic saving paradigm in which human subjects could form internal strategies to save flavored liquid rewards that accumulated with interest; subjects later executed their strategies through choice sequences of self-defined lengths. Experimental manipulation of reward type and interest rate elicited individual differences in saving strategies. We used primary rewards because they elicit distinct subjective preferences [20] and related activations in human reward and decision systems [11, 19, 33], and because they induce “visceral temptations” that promote variation in saving behavior, as shown in previous experimental studies of real-life saving decisions [3].

We observed prospective amygdala activations that predicted subjects’ internal saving strategies up to two minutes before their behavioral completion. This prospective activity encoded two crucial planning components: the number of forthcoming choice steps implied by the current saving strategy, and their subjective evaluation. Amygdala planning activity was functionally coupled to specific prefrontal areas that encoded distinct planning components and reflected individual differences in strategy formation and saving performance. These findings suggest a previously unrecognized planning function for the human amygdala and identify neural components for simple economic saving strategies in functionally coupled amygdala-prefrontal reward circuits.

## Results

### Economic Saving Task

**Figure 1:**
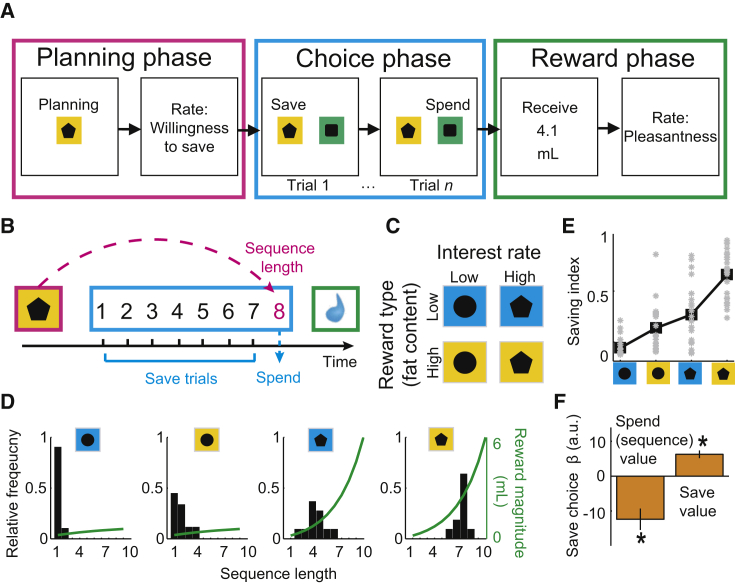
Economic Saving Task and Behavior (A) Subjects planned and performed choice sequences of self-defined lengths to save different types of liquid reward that accumulated according to a given interest rate. Each sequence was defined by a combination of offered reward type (high-fat versus low-fat drink) and interest rate (high versus low), constituting a two-by-two factorial design. The task allowed subjects to plan their behavior up to 2 min in advance (up to 10 consecutive save choices with ∼13 s cycle time, following the ∼13 s planning phase). Randomized left-right positions of the save and spend cues on each trial precluded planning of action sequences. (B) Example saving sequence in which the subject spent on the eighth trial. (C) Experimental conditions and pre-trained cues. (D) Saving behavior in a representative subject. Bars show relative frequencies with which the subject produced different choice sequences. Green curves show reward magnitude increases over sequential save choices. (E) Saving behavior across subjects. The graph shows saving index (based on mean sequence lengths) for individual subjects (gray) and mean across subjects (black). Subjects saved longer (higher saving index) when interest and fat content were high. (F) Saving behavior modeled by subjective values. The graph shows a logistic regression of trial-by-trial save-spend choices on current sequence value (i.e., the value associated with spending on the current trial, derived from choice frequencies [D]) and save value (i.e., the average value of spending on any remaining trial of that sequence) (both p < 0.001, t test; current sequence value; t(23) = −9.64; save value t(23) = 4.55). Error bars represent SEM. See also Figures S1–S3.

Healthy volunteers (n = 24) performed choice sequences of self-defined lengths to save (accumulate) primary rewards (flavored dairy drinks) before choosing to spend (consume) the accumulated rewards (Figures 1A–1C). A sequence began with the planning phase (Figures 1A and 1B), in which pre-trained cues signaled current interest rate and reward type (Figure 1C), allowing subjects to form an internal saving strategy toward a specific reward goal. Subjects then entered the choice phase, in which they progressed toward their goal by making sequential, trial-by-trial save versus spend choices. Following a spend choice, computer-controlled pumps delivered the saved reward. Throughout each sequence, current trial position and saved reward amount were not cued, requiring subjects to track progress internally. Importantly, as learned in a training session, subjects could not influence the occurrence of reward type and interest rate conditions over consecutive sequences. This task design allowed subjects to autonomously plan their behavior within a saving sequence up to 2 min in advance (up to 10 consecutive save choices with ∼13 s cycle time, following the ∼13 s planning phase).

### Saving Behavior and Subjective Value Model

Saving behavior, measured by observed choice sequence lengths, depended on current reward type, current interest rate, and their interaction (Figures 1D and 1E; all p < 0.005, multiple regression). Subjects generally saved longer with higher interest rates and with the high-fat reward type (Figure 1E). Crucially, changes in reward type and interest rate produced substantial variation in saving behavior, both between subjects (Figure 1E, gray dots) and within subjects (Figure 1D; Figure S1), which confirmed the importance of subjective preferences in the present task.

As economic choices critically depend on the subjective values individuals derive from the choice options, we estimated the value of each saving sequence (“sequence value”) from observed choice frequencies (see Supplemental Experimental Procedures). These subjective values depended on final reward amounts and current reward type but also on expenditure related to sequence length. As higher reward amounts required longer sequences (determined by current interest rate), the value of the sequence was compromised by temporal delay and physical effort. To capture these influences on value in a direct manner, we followed the general notion of standard economic choice theory and estimated subjective values from observed behavioral choices. We assumed that a saving sequence had a higher subjective value if the subject chose it more frequently. Values derived in this manner provided a suitable description of the observed saving choices, as confirmed by logistic regression (Figure 1F; Figure S2A; across-subjects pseudo-R^2^ = 0.62 ± 0.02), out-of-sample validation (Figure S2A, inset), correlation with stated saving intentions (R = 0.33, p < 0.001), and correlation with subjects’ bids for the same reward in a separate, auction-like mechanism (Becker-DeGroot-Marschak [BDM] [34]; R = 0.39, p < 0.001). Notably, subjective values provided a better description of subjects’ choices than the objective factors reward type and interest rate, or their interaction (Figure S2). Response times were related to subjective values, differed significantly between save and spend choice trials, and depended on the forthcoming sequence length (Figure S2), consistent with internally planned saving. Furthermore, while subjects approximated objectively optimal decisions in the low-fat/low-interest condition (maximizing rate of reward return, i.e., liquid per trial), they deviated from optimality in other conditions, with substantial inter-subject variation (Figure S3). This further suggested that behavior was guided by subjective valuations of factors reward type and interest rate. Behavior in the current sequence did not depend on the length of the previous sequence (p > 0.05, multiple regression), which confirmed that subjects treated sequences as independent.

Taken together, the combination of reward and interest rate that defined each choice sequence elicited subjective valuations of that sequence, which guided saving behavior.

### Prospective Amygdala Activity Related to Internal Saving Strategies

Classically, the amygdala is associated with affective responses to immediate sensory events [35, 36] rather than internally driven behavioral strategies. Such cue reactivity is also a dominant theme in current views of human amygdala function [37, 38, 39, 40]. By contrast, recent neurophysiological investigations implicate the amygdala in more complex, sequential decision-making [15, 16]. We therefore investigated whether activity in the human amygdala reflected the key strategy components that guided subjects’ saving decisions.

**Figure 2:**
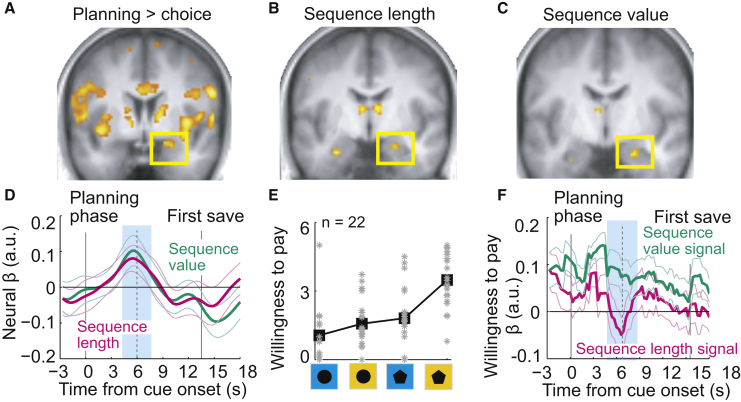
Amygdala Planning Activity Reflects Economic Saving Strategies (A) Stronger amygdala activity in the planning phase compared to the choice phase (cluster p values corrected for family-wise error across the whole brain; map thresholded at p < 0.005, uncorrected for display purposes, extent threshold ≥ 10 voxels). (B) Amygdala activity in the planning phase predicted the length of the forthcoming choice sequence (p < 0.05, small volume correction). (C) Amygdala activity in the planning phase reflected sequence value, i.e., the subjective value of the forthcoming choice sequence (p < 0.05, small volume correction). Sequence value was derived from observed choice frequencies for different saving sequences. (D) Region-of-interest analysis. The graph shows a regression of amygdala activity on sequence length and sequence value. Both factors explained significant variance (p < 0.05, random-effects multiple linear regression; sequence length t(23) = 2.43; sequence value t(23) = 2.45). Neural βs indicate mean regression weights from fitting a multiple linear regression model containing both sequence length and value regressors to neural activity in each subject. Thin colored lines indicate SEM across subjects. “Planning phase” indicates onset of planning phase (at 0 s); “first save” indicates onset of first save trial in sequence. The blue shaded box indicates the analysis period at the expected delay of the hemodynamic response. (E) Behavior in a separate economic task. Subjects (n = 22) performed an economic auction-like (Becker-DeGroot-Marschak [BDM]) task in which they placed willingness-to-pay bids on the same rewards and choice sequences as in the saving experiment. The mean bids per condition are shown for each subject (gray data points) and means across subjects (black). (F) Amygdala planning activity, measured during the saving task, predicted willingness-to-pay bids in the auction-like task. Only the sequence value signal (green βs, based on sequence value-correlated amygdala activity during the saving task) predicted willingness-to-pay bids (p < 0.05, random-effects multiple linear regression; sequence value signal t(21) = 2.45). See also Figures S4 and S5.

Broadly contrasting neural activity in planning and choice phases identified brain areas previously implicated in cognitive control, decision-making, and motivation (Figure 2A; Table S1, GLM1). However, our most striking finding was future-oriented activity in the amygdala that occurred during the planning phase, even before subjects initiated a saving sequence. This “planning activity” predicted the length of the forthcoming choice sequence, up to 2 min before its completion (Figure 2B, GLM1). It was not explained by simple cue responses or reported saving intentions (Figure S4). Importantly, sequence lengths were self-defined by the subjects, rather than instructed, and only existed as an internal, mental representation during the planning phase. In this sense, the observed correlation between amygdala activity and sequence length suggested that amygdala planning activity “predicted” subsequent behavior. Thus, prospective amygdala activity reflected the length of the internally planned choice sequence, which defined the subjects’ behavioral saving strategy.

We observed a second form of prospective amygdala activity that reflected subjects’ valuations of saving strategies, which is crucial for directing planned behavior toward preferred reward goals [1, 5]. Regressing activity on the subjective value of the forthcoming saving sequence (sequence value derived from observed choices) revealed a selective effect in the amygdala (Figure 2C, GLM2), distinct from encoding of planned sequence length (Figure 2D). Importantly, by varying the experimental factors reward type and interest rate, we partly decorrelated chosen sequence lengths from associated values (Figures 1D and 1E; Figure S1), which allowed detection of separate neural effects. The prospective valuation activity encoded specifically the value of the currently planned, forthcoming saving sequence, rather than simply reflecting the average value of the condition cue (regressor for mean sequence value of each condition; p = 0.28, t(23) = 1.1). Thus, in addition to encoding planned sequence length, prospective amygdala activity reflected the subjective value of the current saving strategy.

We tested whether these amygdala planning signals predicted behavior also in a different value elicitation mechanism. On separate days, subjects placed bids in an auction-like mechanism (BDM) to indicate their willingness to pay for the same rewards and choice sequences as in the saving task (Figure 2E). Using a multiple-regression approach, we dissected the amygdala’s planning activity, measured in the saving task, by modeling its two distinct planning signals that correlated with the behavioral saving plan (sequence length) and its value (sequence value), respectively. Only the activity component captured by the sequence value regressor also predicted subjects’ BDM bids in the separate task (Figure 2F). Thus, prospective amygdala value signals predicted behavior in a different economic task, suggesting a flexible economic valuation mechanism.

**Figure 3:**
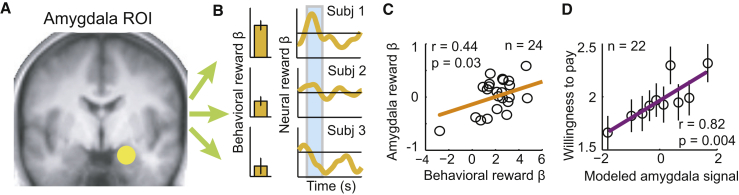
Across-Subject Relation between Amygdala Planning Activity and Individual Differences in Saving Strategy (A) Amygdala region of interest, defined in unbiased manner by leave-one-out method. (B) Illustration of analysis approach: regression of observed sequence lengths on factors reward type and interest rate resulted in subject-specific behavioral βs; a corresponding regression of amygdala activity during the planning phase (extracted from amygdala region of interest) resulted in subject-specific neural βs. Behavioral (left) and neural (right) βs for reward type (“reward β,” reflecting regression coefficients for high-fat versus low-fat content) are shown for three individual subjects. Neural βs are shown as time courses aligned to the onset of the planning phase cue. Blue shaded box indicates analysis period at the expected delay of the hemodynamic response. (C) Neurometric-psychometric comparison across subjects. Behavioral and neural reward βs are plotted for all subjects. Behavioral sensitivity to reward type matched amygdala sensitivity to reward type (significant with robust fit). (D) Amygdala planning activity modeled from individuals’ reward preferences predicted BDM bids. We fitted amygdala activity in the planning phase to reward type, as in (B), and used the resulting model of amygdala planning activity to predict willingness-to-pay bids from the auction-like (BDM) task using linear regression (robust fit). The plot shows means ± SEM for equally populated bins of modeled amygdala activity. This analysis was performed for the 22 subjects for whom BDM data were available. See also Figure S5.

Further analysis investigated relationships between amygdala activity and saving behavior across individual participants. A psychometric-neurometric comparison identified matching sensitivities between individuals’ neural and behavioral measures associated with strategy choice: across individuals, the behavioral influence of factors reward type and interest rate, which determined the choice of saving strategy, matched the neural influence of these factors on amygdala activity (Figures 3A–3C). In other words, individual differences in saving behavior were expressed in the integration of different strategic factors, and amygdala planning activity reflected this integration. Consistently, a model of amygdala planning activity that incorporated these subjective integrations also predicted willingness-to-pay bids elicited in a separate task (Figure 3D). Thus, amygdala planning activity correlated well with individual differences in saving behavior.

Taken together, these data suggest that prospective amygdala activity in the planning phase encoded two crucial components of economic saving strategies [1, 2]: the number of forthcoming choice steps that define the subject’s behavioral saving strategy, and the subjective value that reflects the strategy’s focus on reward.

### Frontal Planning Activities and Functional Connectivity in the Planning Phase

**Figure 4:**
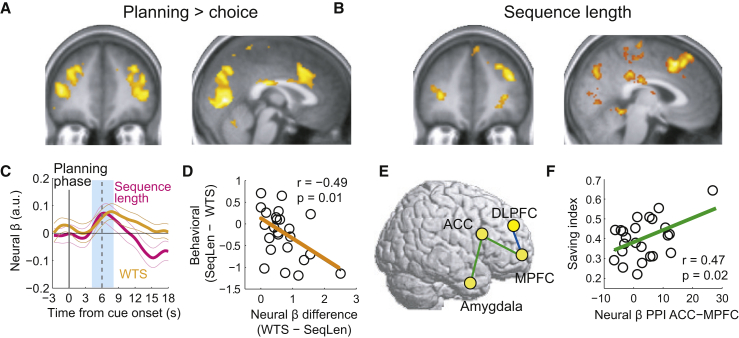
Frontal Cortex Encoding of Saving Strategies and Planning Connectivity (A) Activity in DLPFC and ACC was stronger in the planning phase compared to the choice phase (cluster p values corrected for family-wise error across the whole-brain, p < 0.05; map thresholded at p < 0.005, uncorrected for display purposes, extent threshold ≥ 10 voxels). (B) Activity in DLPFC and ACC in the planning phase predicted the length of the forthcoming choice sequence (p < 0.05, whole-brain correction). (C) Region-of-interest analysis. Planning activity in DLPFC was explained by both reported saving intentions (willingness to save, WTS) and sequence length (p < 0.05, random-effects multiple linear regression; WTS t(23) = 2.6; sequence length t(23) = 2.32). (D) Across subjects, DLPFC coding differences between stated (WTS) and executed (sequence length) saving strategies were related to behavioral deviations from saving intentions (significant with robust fit). (E) Functional connectivity patterns during the planning phase. PPI analyses revealed correlated activity between amygdala and ACC depending on current reward type (high-fat > low-fat content; uncorrected at p = 0.005) and between MPFC and ACC depending on current interest rate (high > low interest rate; p < 0.05, whole-brain correction). Both connectivity patterns were related across subjects (R = 0.46, p = 0.02, significant with robust fit). DLPFC showed stronger coupling with MPFC during the planning phase compared to the choice phase (blue; p < 0.05, whole-brain correction). (F) Across subjects, stronger planning connectivity between ACC and MPFC was related to higher saving performance (significant with robust fit on saving index, derived from mean sequence lengths). See also Figures S2 and S5.

The observed involvement of human amygdala in economic planning required comparisons to prefrontal cortex regions with well-established roles in cognitive control and decision-making [11, 28, 29, 30, 31, 32]. Similar to amygdala, the dorsolateral prefrontal cortex (DLPFC) and anterior cingulate cortex (ACC) were more active during the planning phase than during the choice phase (Figure 4A, GLM1), and their activity predicted the forthcoming number of choice steps (Figure 4B, GLM1). However, neither area reflected the value of the planned saving strategy (nor individual reward preferences; Figure S5). Thus, these frontal areas partly resembled the amygdala by encoding subjects’ behavioral saving strategies (sequence length), but they did not encode initial strategy valuations (sequence value).

Because DLPFC activity is involved in behavioral intentions and information maintenance [41], we tested whether it encoded subjects’ saving intentions in addition to behaviorally executed plans. In the planning phase, DLPFC activity also correlated with subjects’ initially stated willingness to save (WTS; Figure 4C), which suggested joint encoding of intended and executed saving strategies. Reported and executed strategies often corresponded, but subjects also frequently deviated from their stated intentions, which allowed detection of separate neural effects (Figure S2D). These deviations were not random but were partly explained by a combination of objective task factors, subjective valuations, and planning activity in DLPFC (but not ACC or amygdala; Figure S2F). Consistent with these results, discrepant DLPFC coding strengths for stated and executed strategies were related to subjects’ behavioral deviations from stated strategies (Figure 4D).

Frontal cortex planning activities not only resembled amygdala planning activity, they were also functionally coupled to it (Figure 4E; Table S5; Supplemental Experimental Procedures GLM PPI 1-3). Psychophysiological interaction (PPI) analysis in the planning phase with amygdala as seed region identified functional connectivity with ACC. This connection depended on reward type in the current sequence, with enhanced amygdala-ACC connectivity for the typically preferred high-fat rewards compared to low-fat rewards. We found similar connectivity between ACC and another region with known decision functions, the medial prefrontal cortex (MPFC) [10, 11, 28, 30, 31], with enhanced connectivity for high interest rates, which overall elicited longer saving sequences. The strengths of these two functional connections—reward-dependent amygdala-ACC coupling and interest-dependent ACC-MPFC coupling—were correlated across subjects (R = 0.45, p = 0.029), which provided evidence for interacting amygdala-frontal planning activities. Functional connectivity related to interest rate between ACC and MPFC was also stronger in individuals with higher average tendency to save (Figure 4F; performance assessed by saving index, see Figure 1E) and reflected the extent to which subjects approximated rate of reward return (Figure S3H). Together, the relationships to individual differences suggested behavioral relevance for these functional connections. Thus, the formation of simple economic saving strategies engaged functional circuits involving the amygdala and distinct frontal areas.

### Amygdala-Prefrontal Activities during the Choice Phase

**Figure 5:**
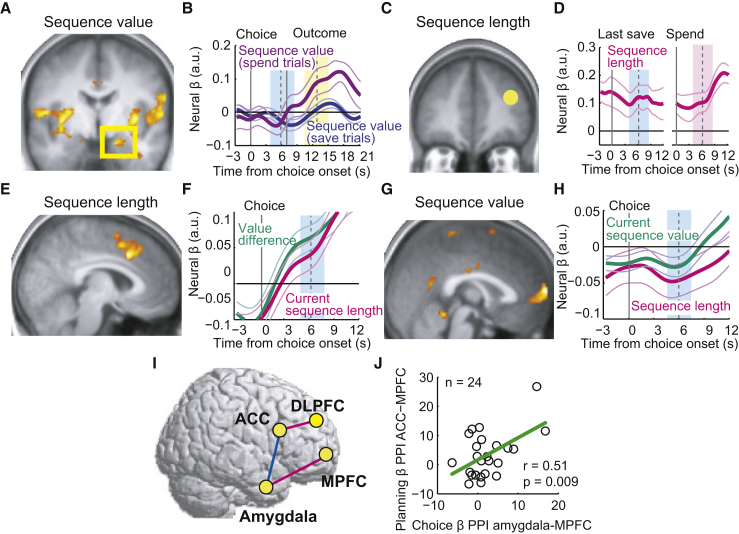
Planning Signals and Functional Connectivity in the Choice Phase (A) Amygdala choice-phase activity correlated with current sequence value (p < 0.05, whole-brain corrected). (B) Amygdala activity reflected the dynamically evolving sequence value during save choices (blue shading; p < 0.05, random-effects multiple linear regression; t(23) = −2.21; negative βs indicate lower activity with higher value). On spend trials, activity encoded sequence value during the reward phase, likely reflecting reward expectation (yellow shading; p < 0.05, random-effects multiple linear regression; t(23) = 2.79; positive β following outcome). (C and D) DLPFC choice-phase activity correlated with planned sequence length (region-of-interest analysis, p < 0.05, random-effects multiple linear regression), specifically during last save choice (blue shading; t(23) = 2.89) and subsequent spend choice (pink shading; t(23) = 4.74). (E) ACC choice-phase activity tracked current position in the sequence, i.e., current sequence length (p < 0.05, whole-brain correction). (F) ACC activity reflected both current sequence length and save-spend value difference (p < 0.05, random-effects multiple linear regression; value difference: t(23) = 3.91; sequence length: t(23) = 2.24). (G) MPFC choice-phase activity correlated with planned sequence value (p < 0.05, whole-brain correction). (H) MPFC activity reflected planned sequence length and current sequence value (p < 0.05, random-effects multiple linear regression; sequence value: t(23) = −2.08; sequence length: t(23) = −2.12; negative βs indicate lower activity with higher value and longer sequences). (I) Functional connectivity patterns during the choice phase. PPI analysis (p < 0.05, whole-brain correction) showed correlated choice-dependent activity (save > spend choice) between amygdala and MPFC and between ACC and DLPFC. Amygdala and ACC had stronger correlated activity during the choice phase compared to the planning phase (blue). (J) Amygdala-MPFC choice-phase connectivity across subjects correlated with planning-phase ACC-MPFC connectivity (significant with robust fit). See also Figure S5.

The same amygdala-prefrontal areas continued to signal saving strategies in the choice phase. Amygdala choice-phase activity was higher for save compared to spend choices (Figure S4), tracked subjective reward rate throughout the experiment (Figure S4), and signaled the momentary value of the current sequence that evolved dynamically over consecutive save choices (“current sequence value”; Figures 5A and 5B). On spend trials, this sequence value signal extended into the outcome phase (Figure 5B, yellow rectangle), potentially reflecting reward expectation [17, 25]. Notably, sensitivity to value in the amygdala’s initial planning activity (Figure 2D) did not match this later outcome-related value signal (across-subjects correlation of neural betas derived from region-of-interest analysis; R = 0.06, p = 0.77). This suggested that sequence value coding in the planning phase did not simply reflect amygdala reward expectation.

Different from the planning phase, choice-phase amygdala activity failed to signal the number of saving steps implied by the current strategy (sequence length). By contrast, the DLPFC planning signal related to forthcoming sequence length reoccurred during choices (Figures 5C and 5D), consistent with DLPFC functions in maintaining task-relevant information [42]. The ACC showed a different, dynamic choice step signal that reflected the evolving length of the current saving sequence, increasing with each further save choice (“current sequence length”; Figure 5E, GLM4). Such progress monitoring is critical for the execution of planned behaviors including economic saving [1, 2, 3] and also occurs in monkey ACC neurons during behavioral sequences [43]. Importantly, ACC progress signals were distinct from known ACC value signals during decision-making [30, 31, 32], which we observed separately (Figure 5F). Finally, signals for planned sequence length and current sequence value converged in MPFC (Figures 5G and 5H), which therefore integrated a maintained sequence length signal with the sequence’s dynamically evolving value. Thus, during both planning and sequential choices, amygdala-prefrontal areas encoded the planning components sequence value and sequence length, which were essential (Figure 1F) for guiding subjects’ saving behavior.

As in the planning phase, we observed functional connectivities between amygdala and prefrontal cortex in the choice phase (Figure 5I; Table S5; GLM PPI1, 4). Specifically, areas that jointly encoded the same planning variable were also functionally connected with each other (Figure 5I, magenta). Choice-dependent coupling (enhanced in save compared to spend choices) occurred between amygdala and MPFC, reflecting their common sequence value signals (GLM PPI4). By contrast, enhanced coupling between ACC and DLPFC during choices (compared to planning) reflected their common sequence length signals (GLM PPI1). These distinct functional connections were linked by a direct, choice-dependent amygdala-ACC connection (Figure 5I, blue, GLM PPI1). Across subjects, specific connection strengths in the choice phase correlated with connection strengths during the planning phase (Figure 5J). These results provided further evidence for functional amygdala-prefrontal circuits that support both saving strategy formation and execution.

## Discussion

Our results suggest that the human amygdala—traditionally associated with emotional reactions to external events—participates in the formation and execution of economic saving strategies toward future rewards. Amygdala planning activity encoded the two key strategy components that guided subjects’ behavior: the length and value of the planned saving sequence. Sequence length signals reflected subjects’ internal behavioral plan by predicting the forthcoming number of saving steps even before subjects initiated a sequence. Sequence value signals reflected subjects’ valuations of planned sequences and predicted economic behavior in a different task on a different testing day, suggesting a flexible, prospective valuation mechanism. Using a whole-brain imaging technique enabled us to identify functional networks associated with the formation and execution of saving strategies. Beyond the amygdala, these networks involved specific frontal areas previously implicated in decision-making, which encoded distinct strategy components and reflected individuals’ saving performance. Taken together, the identified amygdala-frontal planning activities and their functional interactions represent a potential substrate for linking future-oriented economic valuations to internal saving strategies and their behavioral execution.

Strategic saving involves the formation of an internal saving plan motivated by the prospect of future reward, and subsequent plan execution [1]. The observed two components of neural planning activity, related to the length and value of the forthcoming saving sequence, seem to contribute to this process in two ways. First, sequence length signals in amygdala, DLPFC, and ACC encoded the abstract behavioral implication of the current saving strategy; in other words, they signaled the choice of a specific saving plan. They did not reflect action planning, which was precluded by randomized choice cue positions. During plan execution, these signals could help to align sequential choices with the current strategy and provide input to well-characterized motor planning systems in frontal cortex [7] that translate abstract saving intentions into concrete actions. Second, sequence value signals, a specific component of amygdala planning activity, encoded the current strategy’s economic value. Although they occurred time-locked in response to condition cues, they did not reflect generalized cue responses, average cue value, or basic reward expectation. Instead, they conveyed the specific value of the internally planned sequence. Experimental manipulation of both reward type and interest rate led participants to assign different subjective values to identical sequence lengths, depending on the current reward-interest combination. This allowed detection of separate neural signals related to sequence value and length. Sequence value signals likely reflected the subjective value of a sequence that integrated both reward value and cost due to temporal delay and effort, although our experiment was not designed to separately test these value components. The amygdala’s sequence value signal also reflected inter-individual valuation differences and predicted behavior in the separate auction-like BDM task. Such prospective, mechanism-independent valuation of behavioral plans seems suited to inform the initial decision to select a preferred saving strategy and to regulate motivation during subsequent goal pursuit. Encoding of the two planning components likely depended on amygdala-frontal functional interactions, which reflected current parameters for strategy selection and explained variation in saving performance.

During execution of subjects’ saving strategies, the amygdala and functionally coupled MPFC continually evaluated the current sequence and exhibited choice-dependent functional coupling. Such dynamic, sequential valuations in amygdala and MFPC could inform stepwise decision-making according to an internal saving plan. This interpretation is supported by previously described valuation activities in amygdala and MPFC [11, 18, 19, 20, 24, 25, 26, 28, 30, 31] and the deleterious effects of damage to either area on value-guided behavior [23, 44]. Given the amygdala’s outputs to autonomic effectors [37], its sequential valuation could also serve to regulate motivation and affective state in the pursuit of reward goals.

The DLPFC, an area implicated in cognitive planning [29], was more active during planning than choice, encoded both the length of the forthcoming behavioral sequence and subjects’ reported saving intentions, and reflected behavioral deviations from stated intentions. Unlike the amygdala, DLPFC did not encode sequence value, which limits its role in prospective valuation. During the choice phase, DLPFC’s sequence length signal reoccurred specifically on final save trials, when strategy completion was imminent, and lasted until the subsequent spend choice. Although consistent with a general role in planning and maintaining task goals [41], these results identify previously unrecognized DLPFC functions in economic saving.

The ACC is implicated in cognitive control during sequential behaviors [31, 32, 43, 45]. We found that during the choice phase, a dorsal ACC region tracked the progress of subjects’ internally defined saving strategy. This tracking function reflected an internal evaluation, as our task did not offer external progress cues. It was also not explained by commonly reported, separately observed ACC value difference signals [31]. Strikingly, during the planning phase, we found prospective ACC activity not previously characterized, which reflected subjects’ planned sequence length. This suggests that ACC, together with functionally coupled amygdala and DLPFC, contributes to the formation of a saving strategy based on economic valuations. Our main planning variables differ markedly from ACC value signals observed in sequential foraging tasks, which reflect the average value of the foraging environment [31]. Planning signals for sequence length and sequence value specifically reflected the planned, forthcoming course of action and thus seem linked to situations that allow the formation of internal plans multiple steps in advance, as in economic saving. By contrast, the choice phase of our saving task shares elements with foraging. For example, the observed encoding of value difference between save and spend choices in ACC (Figure 5F) is consistent with ACC valuation of current and alternative courses of action [31]. Valuation processes involved in foraging and exploration decisions, which engage similar brain systems to those identified here [31, 46], likely play additional roles in economic saving.

Previous studies identified frontal-subcortical activities underlying cognitive planning [42], model-based learning [13, 14], and prospective imagination [9], which represent important components of reward-guided behavior. Our experiments focused on economic saving strategies defined by the internal formation of a subjectively preferred reward goal [1, 2, 3] and its behavioral pursuit through self-defined choices [1]. By modeling both the formation and execution of saving strategies [1], our experiments necessarily focused on shorter timescales of up to two minutes. We suggest that the presently observed planning signals reflect a basic mechanism engaged by the formation of a behavioral strategy toward future reward. Additional mechanisms likely mediate planned behavior over longer periods, including episodic prospection [12], valuation of effort and persistence [32], and discounting of long-term delayed rewards [10, 11].

The use of primary, liquid rewards to elicit behavioral variation follows previous neuroimaging [11] and behavioral saving experiments [3]. This, together with manipulation of both reward type and interest rate, allowed us to identify neural planning signals related to behaviorally well-characterized subjective valuations. Although valuations for different reward types typically involve overlapping neural circuits [47, 48], future studies will have to confirm planning signals in saving behavior toward abstract, monetary rewards.

We designed our saving task to capture basic components of everyday choice scenarios, such as contributions to a savings account or short-term consumption decisions [1, 2, 3]. Such decisions are subject to continuous temptations to spend or consume accumulated rewards. Similarly, subjects could internally plan their saving behavior but subsequently change their mind during sequence execution, as implied by models of quasi-hyperbolic temporal discounting [11]. Future studies could adapt our paradigm to investigate relationships between saving behavior, inter-temporal preferences [10, 11], and individual commitment attitudes. Furthermore, longitudinal designs and real-life savings data could test links between the presently identified neural mechanisms and individuals’ financial status.

Classical concepts of amygdala function focus on its immediate responses to affective cues [35, 36], whereas current views extend this cue reactivity to complex human behaviors [35, 37, 40]. However, the future-focused economic planning signals demonstrated here are not anticipated by either classical or current concepts. Amygdala planning signals reflected future saving goals well before they were obtained, persisted over sequential choices, and differed from separate basic reward expectation signals following a spend choice. Accordingly, amygdala planning signals differed from known amygdala processing of externally cued, immediate rewards [17, 18, 19, 20, 40] and decision parameters in isolated, single-trial choices [23, 24, 27]. Thus, our data significantly expand current views by demonstrating amygdala sensitivity to internal behavioral strategies and their subjective values.

Interpretation of the present human imaging results is greatly facilitated by detailed evidence about the functional properties of single amygdala neurons, available from monkey experiments in a similar reward-saving task [15, 16]. With the spatial resolution of fMRI, we cannot determine whether sequence value and sequence length signals are separated at single-neuron level (with a typical fMRI voxel containing as many as 5.5 million neurons [49]). However, this is a critical issue for understanding the neural computations involved in selecting a saving strategy. Our monkey studies show that the primate amygdala indeed contains separate but anatomically intermingled neurons encoding the value and length of economic choice sequences [15, 16]. The presently observed amygdala signals likely reflect the activity of these two separate neuronal populations. The location of our main effects is consistent with basolateral and centromedial amygdala, where intermingled sequence value and sequence length neurons are found in monkeys [15, 16]. The coexistence of these signals in the same brain system—shown here for the first time in the human amygdala—might indicate local conversion from economic valuations to behavioral strategies [15, 16], potentially via competitive, inhibitory interactions among neighboring neurons. This process most likely involves frontal areas with known decision functions, which depend on interactions with amygdala [21, 22].

Compared to the monkey studies, the present human experiments provide several new insights. The currently reported planning signals in the human amygdala integrated multiple factors in the subjective valuation of saving plans, including interest rate and reward type. The present data also link amygdala planning activities to a sophisticated, perhaps human-specific form of economic behavior involving the formulation of bids in an auction-like (BDM) mechanism. Critically, we demonstrate that the amygdala’s planning activity and amygdala-frontal connections partly explain inter-individual differences in saving behavior, which relates to key economic issues affecting individuals and societies [3]. Using whole-brain imaging allowed us to uncover functionally connected systems in frontal cortex beyond amygdala with previously unknown functions in economic saving. These frontal areas encode partly distinct planning components and thus represent interesting targets for future single-neuron recordings. Notably, the same amygdala-frontal circuits are implicated in deregulated reward expectation and affective disorders [50], which impact on the motivation to plan for future rewards and pursue distant goals. Our experimental approach to the neurobiology of economic saving could help understand dysfunctional planning and decision functions of amygdala-frontal circuits in such conditions.

### Conclusions

Theories of planned behavior identify a two-stage process that distinguishes initial plan formation from subsequent execution, and a valuation component that directs behavioral strategies toward future rewards. The present data characterize the neural mechanisms underlying these processes during the formation and pursuit of simple economic saving strategies. Our findings suggest an extended view of the human amygdala that includes a planning function for future rewards embedded within prefrontal circuits with distinct planning and decision functions.

## Author Contributions

L.Z., F.G., and W.S. designed the research. L.Z performed experiments. L.Z. and F.G. analyzed the data. L.Z., F.G., and W.S. wrote the manuscript.

## Supplemental Information

Document S1. Figures S1–S5, Tables S1–S5, and Supplemental Experimental Procedures

Document S2. Article plus Supplemental Information
