# Supplementary material for: Neural Basis for Economic Saving Strategies in Human Amygdala-Prefrontal Reward Circuits
Source: Curr Biol. 2016 Nov 21;26(22):3004–13. doi: 10.1016/j.cub.2016.09.016 (PMC5130697; doi:10.1016/j.cub.2016.09.016)
Supplement: Document S2. Article plus Supplemental Information [file mmc2.pdf]

Current Biology

Article

Neural Basis for Economic Saving Strategies in Human Amygdala-Prefrontal Reward Circuits

Highlights

- Neural activity was measured during planning and execution of economic saving
- Amygdala activity predicted the planned number of saving steps and their value
- Amygdala-frontal circuits coded plan components and individual saving differences
- The results suggest a new function for the human amygdala in economic planning

Authors

Leopold Zangemeister, Fabian Grabenhorst, Wolfram Schultz

Correspondence

lz345@cam.ac.uk (L.Z.),
fg292@cam.ac.uk (F.G.)

In Brief

Economic saving is critical for the welfare of individuals and societies. Zangemeister et al. show prospective activities in human amygdala and prefrontal cortex that reflect economic saving plans and individual differences in saving behavior.

Zangemeister et al., 2016, Current Biology *26*, 3004–3013
November 21, 2016 © 2016 The Authors. Published by Elsevier Ltd.
http://dx.doi.org/10.1016/j.cub.2016.09.016

CellPress

Current Biology
Article

Neural Basis for Economic Saving Strategies in Human Amygdala-Prefrontal Reward Circuits

Leopold Zangemeister,[1,2,3,*] Fabian Grabenhorst,[1,2,*] and Wolfram Schultz[1]
[1]Department of Physiology, Development and Neuroscience, University of Cambridge, Downing Street, Cambridge, CB2 3DY, UK
[2]Co-first author
[3]Lead Contact
*Correspondence: lz345@cam.ac.uk (L.Z.), fg292@cam.ac.uk (F.G.)
http://dx.doi.org/10.1016/j.cub.2016.09.016

SUMMARY

Economic saving is an elaborate behavior in which the goal of a reward in the future directs planning and decision-making in the present. Here, we measured neural activity while subjects formed simple economic saving strategies to accumulate rewards and then executed their strategies through choice sequences of self-defined lengths. Before the initiation of a choice sequence, prospective activations in the amygdala predicted subjects' internal saving plans and their value up to two minutes before a saving goal was achieved. The valuation component of this planning activity persisted during execution of the saving strategy and predicted subjects' economic behavior across different tasks and testing days. Functionally coupled amygdala and prefrontal cortex activities encoded distinct planning components that signaled the transition from saving strategy formation to execution and reflected individual differences in saving behavior. Our findings identify candidate neural mechanisms for economic saving in amygdala and prefrontal cortex and suggest a novel planning function for the human amygdala in directing strategic behavior toward self-determined future rewards.

INTRODUCTION

Economic saving is an elaborate form of planned behavior characterized by dynamic, sequential choices and a focus on self-defined future reward [1, 2]. Successful saving is a key determinant of the welfare of individuals and societies, which impacts entire economic systems [3]. Theories in psychology, economics, and reinforcement learning have identified basic principles that underlie planned behaviors involving rewards, such as economic saving: a two-stage process that distinguishes the initial formation of a behavioral strategy from its subsequent execution [1, 4], and a valuation component that directs behavioral strategies toward future rewards [5]. Here, we used fMRI to measure neural activity in an economic reward-saving paradigm that modeled these principles by separating the formation of a reward-based strategy from its execution through sequential choices.

Based on human lesion [6] and neuroimaging evidence and single-cell recordings in monkeys [7], cognitive and action planning are traditionally associated with the frontal lobes. Other prospective functions, such as episodic future thinking and spatial navigation, are associated with medial temporal lobe structures [8, 9]. However, much less is known about how the brain mediates the influence of rewards on planning, despite their crucial importance in directing strategy formation and execution [1, 4, 5]. Studies using intertemporal choice paradigms have uncovered human brain systems for the subjective valuation of delayed rewards [10–12]. More recent investigations of complex multistep reinforcement learning showed that frontal-striatal systems evaluate reward outcomes associated with externally defined choice paths [13, 14]. These studies identified critical neural components for prospective reward valuation but did not address the key features of planned economic saving, which involve the internal construction of a reward-directed strategy and its subsequent execution through choice sequences of self-defined length [1, 3].

Based on recent single-neuron evidence in non-human primates [15, 16], we hypothesized that in the current study the human amygdala would show prospective activity related to subjects' economic saving strategies. Our hypothesis was further motivated by evidence of amygdala functions in basic reward valuation [17–22], processing of economic choice variables [23, 24], and decision-making [25–27]. We also expected the involvement of prefrontal cortex areas, based on their known valuation, cognitive control, and decision functions [11, 28–32].

We designed a sequential economic saving paradigm in which human subjects could form internal strategies to save flavored liquid rewards that accumulated with interest; subjects later executed their strategies through choice sequences of self-defined lengths. Experimental manipulation of reward type and interest rate elicited individual differences in saving strategies. We used primary rewards because they elicit distinct subjective preferences [20] and related activations in human reward and decision systems [11, 19, 33], and because they induce "visceral temptations" that promote variation in saving behavior, as shown in previous experimental studies of real-life saving decisions [3].

We observed prospective amygdala activations that predicted subjects' internal saving strategies up to two minutes before their behavioral completion. This prospective activity encoded two crucial planning components: the number of forthcoming choice steps implied by the current saving strategy, and their

 © 2016 The Authors. Published by Elsevier Ltd.
This is an open access article under the CC BY license (http://creativecommons.org/licenses/by/4.0/).

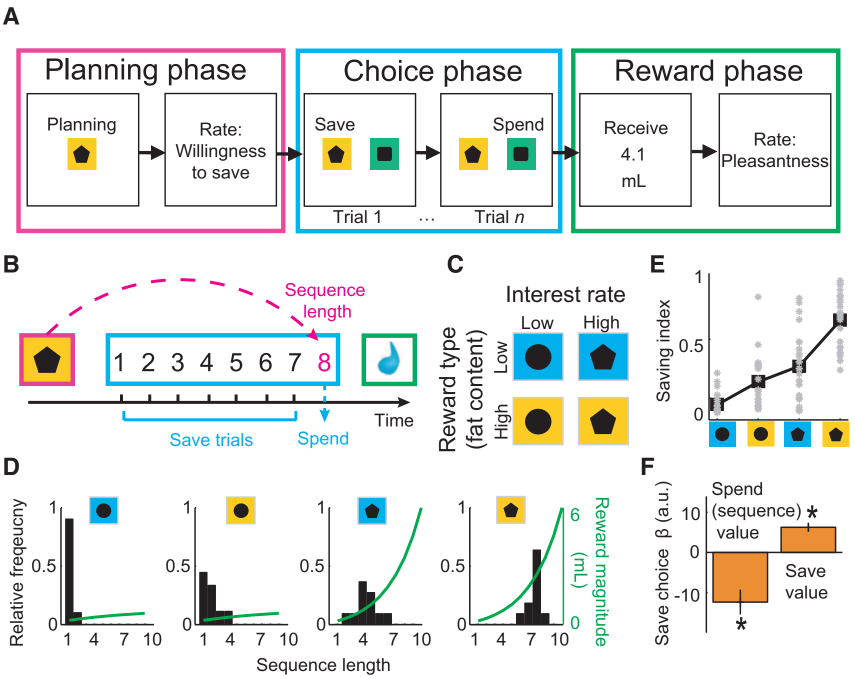

Figure 1. Economic Saving Task and Behavior

(A) Subjects planned and performed choice sequences of self-defined lengths to save different types of liquid reward that accumulated according to a given interest rate. Each sequence was defined by a combination of offered reward type (high-fat versus low-fat drink) and interest rate (high versus low), constituting a two-by-two factorial design. The task allowed subjects to plan their behavior up to 2 min in advance (up to 10 consecutive save choices with ∼13 s cycle time, following the ∼13 s planning phase). Randomized left-right positions of the save and spend cues on each trial precluded planning of action sequences.

(B) Example saving sequence in which the subject spent on the eighth trial.

(C) Experimental conditions and pre-trained cues.

(D) Saving behavior in a representative subject. Bars show relative frequencies with which the subject produced different choice sequences. Green curves show reward magnitude increases over sequential save choices.

(E) Saving behavior across subjects. The graph shows saving index (based on mean sequence lengths) for individual subjects (gray) and mean across subjects (black). Subjects saved longer (higher saving index) when interest and fat content were high.

(F) Saving behavior modeled by subjective values. The graph shows a logistic regression of trial-by-trial save-spend choices on current sequence value (i.e., the value associated with spending on the current trial, derived from choice frequencies [D]) and save value (i.e., the average value of spending on any remaining trial of that sequence) (both $p < 0.001$, t test; current sequence value; $t(23) = -9.64$; save value $t(23) = 4.55$). Error bars represent SEM.

See also Figures S1–S3.

subjective evaluation. Amygdala planning activity was functionally coupled to specific prefrontal areas that encoded distinct planning components and reflected individual differences in strategy formation and saving performance. These findings suggest a previously unrecognized planning function for the human amygdala and identify neural components for simple economic saving strategies in functionally coupled amygdala-prefrontal reward circuits.

RESULTS

Economic Saving Task

Healthy volunteers (n = 24) performed choice sequences of self-defined lengths to save (accumulate) primary rewards (flavored dairy drinks) before choosing to spend (consume) the accumulated rewards (Figures 1A–1C). A sequence began with the planning phase (Figures 1A and 1B), in which pre-trained cues signaled current interest rate and reward type (Figure 1C), allowing subjects to form an internal saving strategy toward a specific reward goal. Subjects then entered the choice phase, in which they progressed toward their goal by making sequential, trial-by-trial save versus spend choices. Following a spend choice, computer-controlled pumps delivered the saved reward. Throughout each sequence, current trial position and saved reward amount were not cued, requiring subjects to track progress internally. Importantly, as learned in a training session, subjects could not influence the occurrence of reward type and interest rate conditions over consecutive sequences. This task design allowed subjects to autonomously plan their behavior within a saving sequence up to 2 min in advance (up to 10 consecutive save choices with ∼13 s cycle time, following the ∼13 s planning phase).

Saving Behavior and Subjective Value Model

Saving behavior, measured by observed choice sequence lengths, depended on current reward type, current interest rate, and their interaction (Figures 1D and 1E; all $p < 0.005$, multiple regression). Subjects generally saved longer with higher interest rates and with the high-fat reward type (Figure 1E). Crucially, changes in reward type and interest rate produced substantial variation in saving behavior, both between subjects (Figure 1E, gray dots) and within subjects (Figure 1D; Figure S1), which confirmed the importance of subjective preferences in the present task.

As economic choices critically depend on the subjective values individuals derive from the choice options, we estimated the value of each saving sequence (“sequence value”) from observed choice frequencies (see Supplemental Experimental Procedures). These subjective values depended on final reward amounts and current reward type but also on expenditure related to sequence length. As higher reward amounts required longer sequences (determined by current interest rate), the value of the sequence was compromised by temporal delay and physical effort. To capture these influences on value in a direct manner, we followed the general notion of standard economic choice theory and estimated subjective values from observed behavioral choices. We assumed that a saving sequence had a higher subjective value if the subject chose it more frequently. Values

derived in this manner provided a suitable description of the observed saving choices, as confirmed by logistic regression (Figure 1F; Figure S2A; across-subjects pseudo-R^2 = 0.62 ± 0.02), out-of-sample validation (Figure S2A, inset), correlation with stated saving intentions (R = 0.33, $p < 0.001$), and correlation with subjects' bids for the same reward in a separate, auction-like mechanism (Becker-DeGroot-Marschak [BDM] [34]; R = 0.39, $p < 0.001$). Notably, subjective values provided a better description of subjects' choices than the objective factors reward type and interest rate, or their interaction (Figure S2). Response times were related to subjective values, differed significantly between save and spend choice trials, and depended on the forthcoming sequence length (Figure S2), consistent with internally planned saving. Furthermore, while subjects approximated objectively optimal decisions in the low-fat/low-interest condition (maximizing rate of reward return, i.e., liquid per trial), they deviated from optimality in other conditions, with substantial inter-subject variation (Figure S3). This further suggested that behavior was guided by subjective valuations of factors reward type and interest rate. Behavior in the current sequence did not depend on the length of the previous sequence ($p > 0.05$, multiple regression), which confirmed that subjects treated sequences as independent.

Taken together, the combination of reward and interest rate that defined each choice sequence elicited subjective valuations of that sequence, which guided saving behavior.

Prospective Amygdala Activity Related to Internal Saving Strategies

Classically, the amygdala is associated with affective responses to immediate sensory events [35, 36] rather than internally driven behavioral strategies. Such cue reactivity is also a dominant theme in current views of human amygdala function [37–40]. By contrast, recent neurophysiological investigations implicate the amygdala in more complex, sequential decision-making [15, 16]. We therefore investigated whether activity in the human amygdala reflected the key strategy components that guided subjects' saving decisions.

Broadly contrasting neural activity in planning and choice phases identified brain areas previously implicated in cognitive control, decision-making, and motivation (Figure 2A; Table S1, GLM1). However, our most striking finding was future-oriented activity in the amygdala that occurred during the planning phase, even before subjects initiated a saving sequence. This "planning activity" predicted the length of the forthcoming choice sequence, up to 2 min before its completion (Figure 2B, GLM1). It was not explained by simple cue responses or reported saving intentions (Figure S4). Importantly, sequence lengths were self-defined by the subjects, rather than instructed, and only existed as an internal, mental representation during the planning phase. In this sense, the observed correlation between amygdala activity and sequence length suggested that amygdala planning activity "predicted" subsequent behavior. Thus, prospective amygdala activity reflected the length of the internally planned choice sequence, which defined the subjects' behavioral saving strategy.

We observed a second form of prospective amygdala activity that reflected subjects' valuations of saving strategies, which is crucial for directing planned behavior toward preferred reward goals [1, 5]. Regressing activity on the subjective value of the forthcoming saving sequence (sequence value derived from observed choices) revealed a selective effect in the amygdala (Figure 2C, GLM2), distinct from encoding of planned sequence length (Figure 2D). Importantly, by varying the experimental factors reward type and interest rate, we partly decorrelated chosen sequence lengths from associated values (Figures 1D and 1E; Figure S1), which allowed detection of separate neural effects. The prospective valuation activity encoded specifically the value of the currently planned, forthcoming saving sequence, rather than simply reflecting the average value of the condition cue (regressor for mean sequence value of each condition; p = 0.28, t(23) = 1.1). Thus, in addition to encoding planned sequence length, prospective amygdala activity reflected the subjective value of the current saving strategy.

We tested whether these amygdala planning signals predicted behavior also in a different value elicitation mechanism. On separate days, subjects placed bids in an auction-like mechanism (BDM) to indicate their willingness to pay for the same rewards and choice sequences as in the saving task (Figure 2E). Using a multiple-regression approach, we dissected the amygdala's planning activity, measured in the saving task, by modeling its two distinct planning signals that correlated with the behavioral saving plan (sequence length) and its value (sequence value), respectively. Only the activity component captured by the sequence value regressor also predicted subjects' BDM bids in the separate task (Figure 2F). Thus, prospective amygdala value signals predicted behavior in a different economic task, suggesting a flexible economic valuation mechanism.

Further analysis investigated relationships between amygdala activity and saving behavior across individual participants. A psychometric-neurometric comparison identified matching sensitivities between individuals' neural and behavioral measures associated with strategy choice: across individuals, the behavioral influence of factors reward type and interest rate, which determined the choice of saving strategy, matched the neural influence of these factors on amygdala activity (Figures 3A–3C). In other words, individual differences in saving behavior were expressed in the integration of different strategic factors, and amygdala planning activity reflected this integration. Consistently, a model of amygdala planning activity that incorporated these subjective integrations also predicted willingness-to-pay bids elicited in a separate task (Figure 3D). Thus, amygdala planning activity correlated well with individual differences in saving behavior.

Taken together, these data suggest that prospective amygdala activity in the planning phase encoded two crucial components of economic saving strategies [1, 2]: the number of forthcoming choice steps that define the subject's behavioral saving strategy, and the subjective value that reflects the strategy's focus on reward.

Frontal Planning Activities and Functional Connectivity in the Planning Phase

The observed involvement of human amygdala in economic planning required comparisons to prefrontal cortex regions with well-established roles in cognitive control and decision-making [11, 28–32]. Similar to amygdala, the dorsolateral prefrontal cortex (DLPFC) and anterior cingulate cortex (ACC)

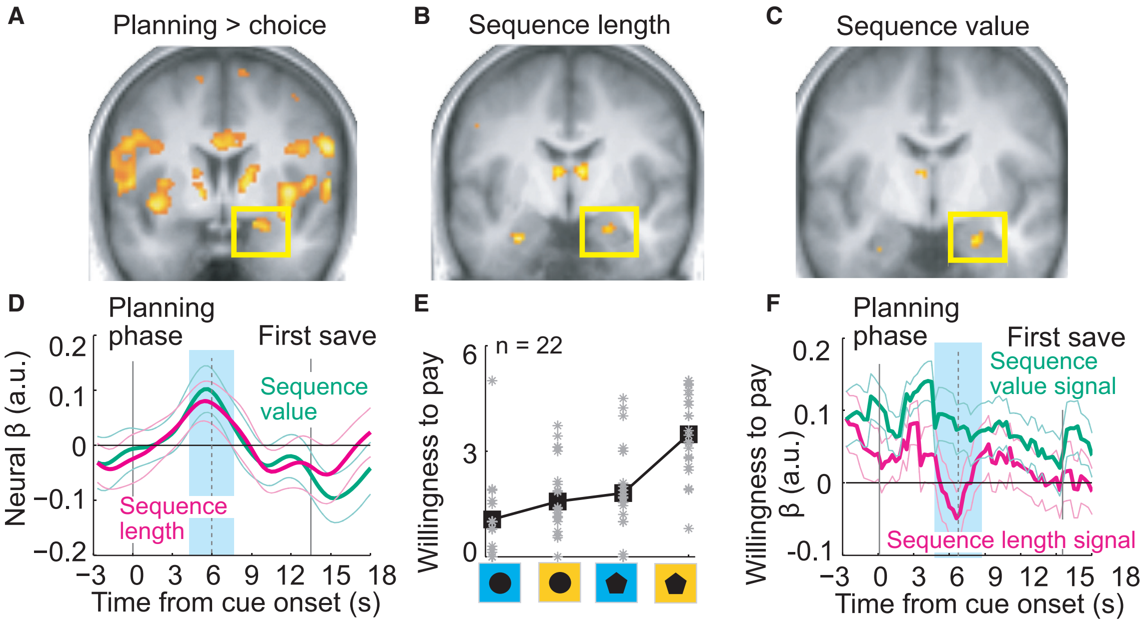

Figure 2. Amygdala Planning Activity Reflects Economic Saving Strategies

(A) Stronger amygdala activity in the planning phase compared to the choice phase (cluster p values corrected for family-wise error across the whole brain; map thresholded at $p < 0.005$, uncorrected for display purposes, extent threshold $\geq$ 10 voxels).

(B) Amygdala activity in the planning phase predicted the length of the forthcoming choice sequence ($p < 0.05$, small volume correction).

(C) Amygdala activity in the planning phase reflected sequence value, i.e., the subjective value of the forthcoming choice sequence ($p < 0.05$, small volume correction). Sequence value was derived from observed choice frequencies for different saving sequences.

(D) Region-of-interest analysis. The graph shows a regression of amygdala activity on sequence length and sequence value. Both factors explained significant variance ($p < 0.05$, random-effects multiple linear regression; sequence length $t(23) = 2.43$; sequence value $t(23) = 2.45$). Neural βs indicate mean regression weights from fitting a multiple linear regression model containing both sequence length and value regressors to neural activity in each subject. Thin colored lines indicate SEM across subjects. “Planning phase” indicates onset of planning phase (at 0 s); “first save” indicates onset of first save trial in sequence. The blue shaded box indicates the analysis period at the expected delay of the hemodynamic response.

(E) Behavior in a separate economic task. Subjects (n = 22) performed an economic auction-like (Becker-DeGroot-Marschak [BDM]) task in which they placed willingness-to-pay bids on the same rewards and choice sequences as in the saving experiment. The mean bids per condition are shown for each subject (gray data points) and means across subjects (black).

(F) Amygdala planning activity, measured during the saving task, predicted willingness-to-pay bids in the auction-like task. Only the sequence value signal (green βs, based on sequence value-correlated amygdala activity during the saving task) predicted willingness-to-pay bids ($p < 0.05$, random-effects multiple linear regression; sequence value signal $t(21) = 2.45$).

See also Figures S4 and S5.

were more active during the planning phase than during the choice phase (Figure 4A, GLM1), and their activity predicted the forthcoming number of choice steps (Figure 4B, GLM1). However, neither area reflected the value of the planned saving strategy (nor individual reward preferences; Figure S5). Thus, these frontal areas partly resembled the amygdala by encoding subjects' behavioral saving strategies (sequence length), but they did not encode initial strategy valuations (sequence value).

Because DLPFC activity is involved in behavioral intentions and information maintenance [41], we tested whether it encoded subjects' saving intentions in addition to behaviorally executed plans. In the planning phase, DLPFC activity also correlated with subjects' initially stated willingness to save (WTS; Figure 4C), which suggested joint encoding of intended and executed saving strategies. Reported and executed strategies often corresponded, but subjects also frequently deviated from their stated intentions, which allowed detection of separate neural effects (Figure S2D). These deviations were not random but were partly explained by a combination of objective task factors, subjective valuations, and planning activity in DLPFC (but not ACC or amygdala; Figure S2F). Consistent with these results, discrepant DLPFC coding strengths for stated and executed strategies were related to subjects' behavioral deviations from stated strategies (Figure 4D).

Frontal cortex planning activities not only resembled amygdala planning activity, they were also functionally coupled to it (Figure 4E; Table S5; Supplemental Experimental Procedures GLM PPI 1-3). Psychophysiological interaction (PPI) analysis in the planning phase with amygdala as seed region identified functional connectivity with ACC. This connection depended on reward type in the current sequence, with enhanced amygdala-ACC connectivity for the typically preferred high-fat rewards compared to low-fat rewards. We found similar connectivity between ACC and another region with known decision functions, the medial prefrontal cortex (MPFC) [10, 11, 28, 30, 31], with enhanced connectivity for high interest rates, which overall elicited longer saving sequences. The strengths of these two functional connections—reward-dependent amygdala-ACC

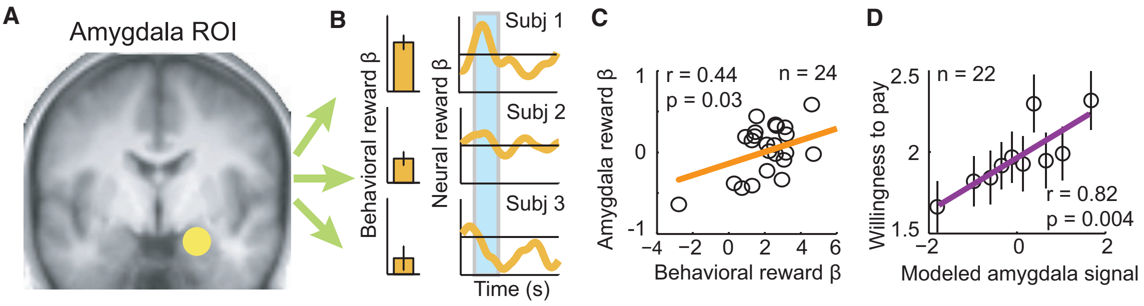

Figure 3. Across-Subject Relation between Amygdala Planning Activity and Individual Differences in Saving Strategy

(A) Amygdala region of interest, defined in unbiased manner by leave-one-out method.

(B) Illustration of analysis approach: regression of observed sequence lengths on factors reward type and interest rate resulted in subject-specific behavioral βs; a corresponding regression of amygdala activity during the planning phase (extracted from amygdala region of interest) resulted in subject-specific neural βs. Behavioral (left) and neural (right) βs for reward type ("reward β," reflecting regression coefficients for high-fat versus low-fat content) are shown for three individual subjects. Neural βs are shown as time courses aligned to the onset of the planning phase cue. Blue shaded box indicates analysis period at the expected delay of the hemodynamic response.

(C) Neurometric-psychometric comparison across subjects. Behavioral and neural reward βs are plotted for all subjects. Behavioral sensitivity to reward type matched amygdala sensitivity to reward type (significant with robust fit).

(D) Amygdala planning activity modeled from individuals' reward preferences predicted BDM bids. We fitted amygdala activity in the planning phase to reward type, as in (B), and used the resulting model of amygdala planning activity to predict willingness-to-pay bids from the auction-like (BDM) task using linear regression (robust fit). The plot shows means ± SEM for equally populated bins of modeled amygdala activity. This analysis was performed for the 22 subjects for whom BDM data were available.

See also Figure S5.

coupling and interest-dependent ACC-MPFC coupling—were correlated across subjects ($R = 0.45$, $p = 0.029$), which provided evidence for interacting amygdala-frontal planning activities. Functional connectivity related to interest rate between ACC and MPFC was also stronger in individuals with higher average tendency to save (Figure 4F; performance assessed by saving index, see Figure 1E) and reflected the extent to which subjects approximated rate of reward return (Figure S3H). Together, the relationships to individual differences suggested behavioral relevance for these functional connections. Thus, the formation of simple economic saving strategies engaged functional circuits involving the amygdala and distinct frontal areas.

Amygdala-Prefrontal Activities during the Choice Phase

The same amygdala-prefrontal areas continued to signal saving strategies in the choice phase. Amygdala choice-phase activity was higher for save compared to spend choices (Figure S4), tracked subjective reward rate throughout the experiment (Figure S4), and signaled the momentary value of the current sequence that evolved dynamically over consecutive save choices ("current sequence value"; Figures 5A and 5B). On spend trials, this sequence value signal extended into the outcome phase (Figure 5B, yellow rectangle), potentially reflecting reward expectation [17, 25]. Notably, sensitivity to value in the amygdala's initial planning activity (Figure 2D) did not match this later outcome-related value signal (across-subjects correlation of neural betas derived from region-of-interest analysis; $R = 0.06$, $p = 0.77$). This suggested that sequence value coding in the planning phase did not simply reflect amygdala reward expectation.

Different from the planning phase, choice-phase amygdala activity failed to signal the number of saving steps implied by the current strategy (sequence length). By contrast, the DLPFC planning signal related to forthcoming sequence length reoccurred during choices (Figures 5C and 5D), consistent with DLPFC functions in maintaining task-relevant information [42]. The ACC showed a different, dynamic choice step signal that reflected the evolving length of the current saving sequence, increasing with each further save choice ("current sequence length"; Figure 5E, GLM4). Such progress monitoring is critical for the execution of planned behaviors including economic saving [1–3] and also occurs in monkey ACC neurons during behavioral sequences [43]. Importantly, ACC progress signals were distinct from known ACC value signals during decision-making [30–32], which we observed separately (Figure 5F). Finally, signals for planned sequence length and current sequence value converged in MPFC (Figures 5G and 5H), which therefore integrated a maintained sequence length signal with the sequence's dynamically evolving value. Thus, during both planning and sequential choices, amygdala-prefrontal areas encoded the planning components sequence value and sequence length, which were essential (Figure 1F) for guiding subjects' saving behavior.

As in the planning phase, we observed functional connectivities between amygdala and prefrontal cortex in the choice phase (Figure 5I; Table S5; GLM PPI1, 4). Specifically, areas that jointly encoded the same planning variable were also functionally connected with each other (Figure 5I, magenta). Choice-dependent coupling (enhanced in save compared to spend choices) occurred between amygdala and MPFC, reflecting their common sequence value signals (GLM PPI4). By contrast, enhanced coupling between ACC and DLPFC during choices (compared to planning) reflected their common sequence length signals (GLM PPI1). These distinct functional connections were linked by a direct, choice-dependent amygdala-ACC connection (Figure 5I, blue, GLM PPI1). Across subjects, specific connection strengths in the choice phase correlated with connection strengths during the planning phase (Figure 5J). These results

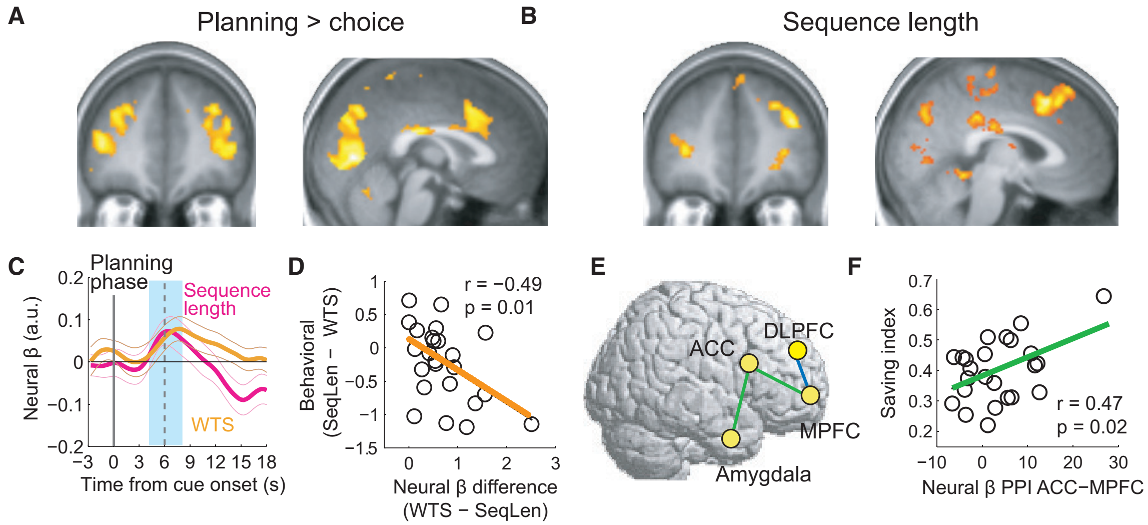

Figure 4. Frontal Cortex Encoding of Saving Strategies and Planning Connectivity

(A) Activity in DLPFC and ACC was stronger in the planning phase compared to the choice phase (cluster p values corrected for family-wise error across the whole-brain, $p < 0.05$; map thresholded at $p < 0.005$, uncorrected for display purposes, extent threshold $\geq$ 10 voxels).

(B) Activity in DLPFC and ACC in the planning phase predicted the length of the forthcoming choice sequence ($p < 0.05$, whole-brain correction).

(C) Region-of-interest analysis. Planning activity in DLPFC was explained by both reported saving intentions (willingness to save, WTS) and sequence length ($p < 0.05$, random-effects multiple linear regression; WTS $t(23) = 2.6$; sequence length $t(23) = 2.32$).

(D) Across subjects, DLPFC coding differences between stated (WTS) and executed (sequence length) saving strategies were related to behavioral deviations from saving intentions (significant with robust fit).

(E) Functional connectivity patterns during the planning phase. PPI analyses revealed correlated activity between amygdala and ACC depending on current reward type (high-fat > low-fat content; uncorrected at $p = 0.005$) and between MPFC and ACC depending on current interest rate (high > low interest rate; $p < 0.05$, whole-brain correction). Both connectivity patterns were related across subjects ($R = 0.46$, $p = 0.02$, significant with robust fit). DLPFC showed stronger coupling with MPFC during the planning phase compared to the choice phase (blue; $p < 0.05$, whole-brain correction).

(F) Across subjects, stronger planning connectivity between ACC and MPFC was related to higher saving performance (significant with robust fit on saving index, derived from mean sequence lengths).

See also Figures S2 and S5.

provided further evidence for functional amygdala-prefrontal circuits that support both saving strategy formation and execution.

DISCUSSION

Our results suggest that the human amygdala—traditionally associated with emotional reactions to external events—participates in the formation and execution of economic saving strategies toward future rewards. Amygdala planning activity encoded the two key strategy components that guided subjects' behavior: the length and value of the planned saving sequence. Sequence length signals reflected subjects' internal behavioral plan by predicting the forthcoming number of saving steps even before subjects initiated a sequence. Sequence value signals reflected subjects' valuations of planned sequences and predicted economic behavior in a different task on a different testing day, suggesting a flexible, prospective valuation mechanism. Using a whole-brain imaging technique enabled us to identify functional networks associated with the formation and execution of saving strategies. Beyond the amygdala, these networks involved specific frontal areas previously implicated in decision-making, which encoded distinct strategy components and reflected individuals' saving performance. Taken together, the identified amygdala-frontal planning activities and their functional interactions represent a potential substrate for linking future-oriented economic valuations to internal saving strategies and their behavioral execution.

Strategic saving involves the formation of an internal saving plan motivated by the prospect of future reward, and subsequent plan execution [1]. The observed two components of neural planning activity, related to the length and value of the forthcoming saving sequence, seem to contribute to this process in two ways. First, sequence length signals in amygdala, DLPFC, and ACC encoded the abstract behavioral implication of the current saving strategy; in other words, they signaled the choice of a specific saving plan. They did not reflect action planning, which was precluded by randomized choice cue positions. During plan execution, these signals could help to align sequential choices with the current strategy and provide input to well-characterized motor planning systems in frontal cortex [7] that translate abstract saving intentions into concrete actions. Second, sequence value signals, a specific component of amygdala planning activity, encoded the current strategy's economic value. Although they occurred time-locked in response to condition cues, they did not reflect generalized cue responses, average cue value, or basic reward expectation. Instead, they conveyed the specific value of the internally planned sequence. Experimental manipulation of both reward type and interest rate led participants to assign different subjective values to identical sequence lengths, depending on the current reward-interest combination. This allowed detection of separate

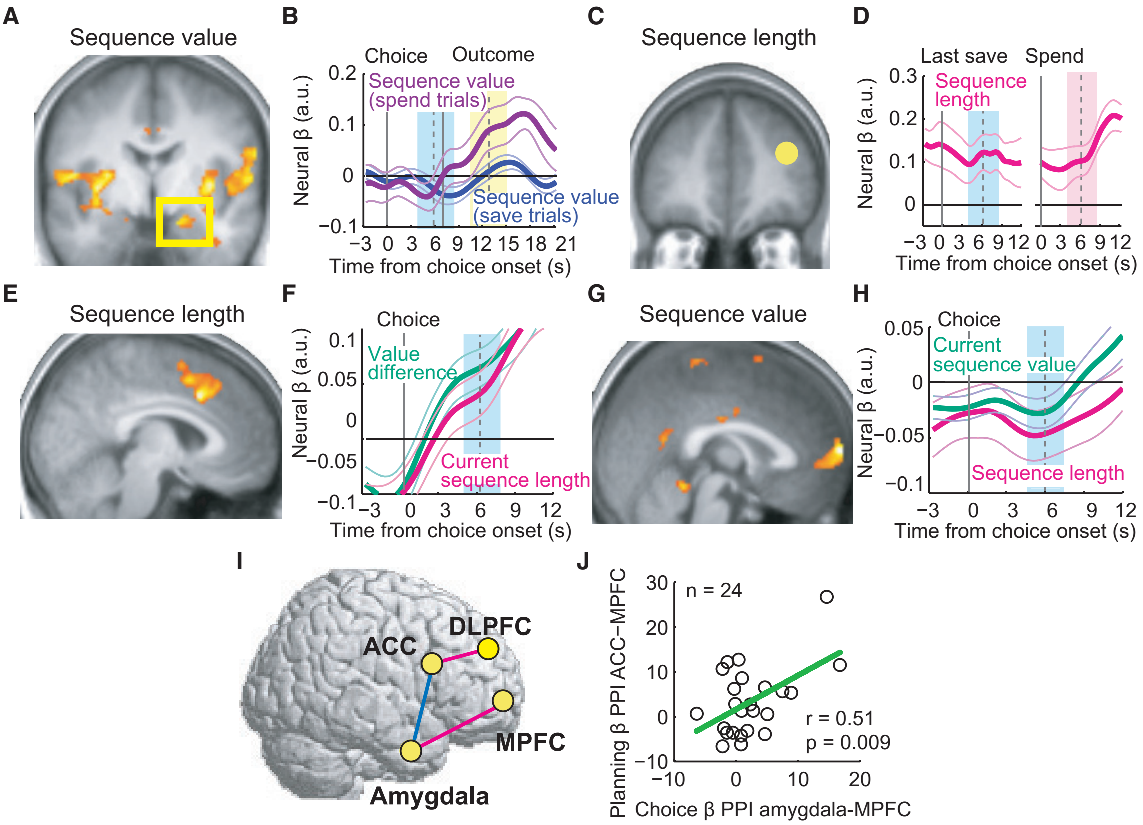

Figure 5. Planning Signals and Functional Connectivity in the Choice Phase

(A) Amygdala choice-phase activity correlated with current sequence value ($p < 0.05$, whole-brain corrected).

(B) Amygdala activity reflected the dynamically evolving sequence value during save choices (blue shading; $p < 0.05$, random-effects multiple linear regression; $t(23) = -2.21$; negative βs indicate lower activity with higher value). On spend trials, activity encoded sequence value during the reward phase, likely reflecting reward expectation (yellow shading; $p < 0.05$, random-effects multiple linear regression; $t(23) = 2.79$; positive β following outcome).

(C and D) DLPFC choice-phase activity correlated with planned sequence length (region-of-interest analysis, $p < 0.05$, random-effects multiple linear regression), specifically during last save choice (blue shading; $t(23) = 2.89$) and subsequent spend choice (pink shading; $t(23) = 4.74$).

(E) ACC choice-phase activity tracked current position in the sequence, i.e., current sequence length ($p < 0.05$, whole-brain correction).

(F) ACC activity reflected both current sequence length and save-spend value difference ($p < 0.05$, random-effects multiple linear regression; value difference: $t(23) = 3.91$; sequence length: $t(23) = 2.24$).

(G) MPFC choice-phase activity correlated with planned sequence value ($p < 0.05$, whole-brain correction).

(H) MPFC activity reflected planned sequence length and current sequence value ($p < 0.05$, random-effects multiple linear regression; sequence value: $t(23) = -2.08$; sequence length: $t(23) = -2.12$; negative βs indicate lower activity with higher value and longer sequences).

(I) Functional connectivity patterns during the choice phase. PPI analysis ($p < 0.05$, whole-brain correction) showed correlated choice-dependent activity (save > spend choice) between amygdala and MPFC and between ACC and DLPFC. Amygdala and ACC had stronger correlated activity during the choice phase compared to the planning phase (blue).

(J) Amygdala-MPFC choice-phase connectivity across subjects correlated with planning-phase ACC-MPFC connectivity (significant with robust fit).

See also Figure S5.

neural signals related to sequence value and length. Sequence value signals likely reflected the subjective value of a sequence that integrated both reward value and cost due to temporal delay and effort, although our experiment was not designed to separately test these value components. The amygdala's sequence value signal also reflected inter-individual valuation differences and predicted behavior in the separate auction-like BDM task. Such prospective, mechanism-independent valuation of behavioral plans seems suited to inform the initial decision to select a preferred saving strategy and to regulate motivation during subsequent goal pursuit. Encoding of the two planning components likely depended on amygdala-frontal functional interactions, which reflected current parameters for strategy selection and explained variation in saving performance.

During execution of subjects' saving strategies, the amygdala and functionally coupled MPFC continually evaluated the current sequence and exhibited choice-dependent functional coupling. Such dynamic, sequential valuations in amygdala and MFPC could inform stepwise decision-making according to an internal saving plan. This interpretation is supported by previously described valuation activities in amygdala and MPFC [11, 18–20, 24–26, 28, 30, 31] and the deleterious effects of damage to either area on value-guided behavior [23, 44]. Given the amygdala's outputs to autonomic effectors [37], its

sequential valuation could also serve to regulate motivation and affective state in the pursuit of reward goals.

The DLPFC, an area implicated in cognitive planning [29], was more active during planning than choice, encoded both the length of the forthcoming behavioral sequence and subjects' reported saving intentions, and reflected behavioral deviations from stated intentions. Unlike the amygdala, DLPFC did not encode sequence value, which limits its role in prospective valuation. During the choice phase, DLPFC's sequence length signal reoccurred specifically on final save trials, when strategy completion was imminent, and lasted until the subsequent spend choice. Although consistent with a general role in planning and maintaining task goals [41], these results identify previously unrecognized DLPFC functions in economic saving.

The ACC is implicated in cognitive control during sequential behaviors [31, 32, 43, 45]. We found that during the choice phase, a dorsal ACC region tracked the progress of subjects' internally defined saving strategy. This tracking function reflected an internal evaluation, as our task did not offer external progress cues. It was also not explained by commonly reported, separately observed ACC value difference signals [31]. Strikingly, during the planning phase, we found prospective ACC activity not previously characterized, which reflected subjects' planned sequence length. This suggests that ACC, together with functionally coupled amygdala and DLPFC, contributes to the formation of a saving strategy based on economic valuations. Our main planning variables differ markedly from ACC value signals observed in sequential foraging tasks, which reflect the average value of the foraging environment [31]. Planning signals for sequence length and sequence value specifically reflected the planned, forthcoming course of action and thus seem linked to situations that allow the formation of internal plans multiple steps in advance, as in economic saving. By contrast, the choice phase of our saving task shares elements with foraging. For example, the observed encoding of value difference between save and spend choices in ACC (Figure 5F) is consistent with ACC valuation of current and alternative courses of action [31]. Valuation processes involved in foraging and exploration decisions, which engage similar brain systems to those identified here [31, 46], likely play additional roles in economic saving.

Previous studies identified frontal-subcortical activities underlying cognitive planning [42], model-based learning [13, 14], and prospective imagination [9], which represent important components of reward-guided behavior. Our experiments focused on economic saving strategies defined by the internal formation of a subjectively preferred reward goal [1–3] and its behavioral pursuit through self-defined choices [1]. By modeling both the formation and execution of saving strategies [1], our experiments necessarily focused on shorter timescales of up to two minutes. We suggest that the presently observed planning signals reflect a basic mechanism engaged by the formation of a behavioral strategy toward future reward. Additional mechanisms likely mediate planned behavior over longer periods, including episodic prospection [12], valuation of effort and persistence [32], and discounting of long-term delayed rewards [10, 11].

The use of primary, liquid rewards to elicit behavioral variation follows previous neuroimaging [11] and behavioral saving experiments [3]. This, together with manipulation of both reward type and interest rate, allowed us to identify neural planning signals related to behaviorally well-characterized subjective valuations. Although valuations for different reward types typically involve overlapping neural circuits [47, 48], future studies will have to confirm planning signals in saving behavior toward abstract, monetary rewards.

We designed our saving task to capture basic components of everyday choice scenarios, such as contributions to a savings account or short-term consumption decisions [1–3]. Such decisions are subject to continuous temptations to spend or consume accumulated rewards. Similarly, subjects could internally plan their saving behavior but subsequently change their mind during sequence execution, as implied by models of quasi-hyperbolic temporal discounting [11]. Future studies could adapt our paradigm to investigate relationships between saving behavior, inter-temporal preferences [10, 11], and individual commitment attitudes. Furthermore, longitudinal designs and real-life savings data could test links between the presently identified neural mechanisms and individuals' financial status.

Classical concepts of amygdala function focus on its immediate responses to affective cues [35, 36], whereas current views extend this cue reactivity to complex human behaviors [35, 37, 40]. However, the future-focused economic planning signals demonstrated here are not anticipated by either classical or current concepts. Amygdala planning signals reflected future saving goals well before they were obtained, persisted over sequential choices, and differed from separate basic reward expectation signals following a spend choice. Accordingly, amygdala planning signals differed from known amygdala processing of externally cued, immediate rewards [17–20, 40] and decision parameters in isolated, single-trial choices [23, 24, 27]. Thus, our data significantly expand current views by demonstrating amygdala sensitivity to internal behavioral strategies and their subjective values.

Interpretation of the present human imaging results is greatly facilitated by detailed evidence about the functional properties of single amygdala neurons, available from monkey experiments in a similar reward-saving task [15, 16]. With the spatial resolution of fMRI, we cannot determine whether sequence value and sequence length signals are separated at single-neuron level (with a typical fMRI voxel containing as many as 5.5 million neurons [49]). However, this is a critical issue for understanding the neural computations involved in selecting a saving strategy. Our monkey studies show that the primate amygdala indeed contains separate but anatomically intermingled neurons encoding the value and length of economic choice sequences [15, 16]. The presently observed amygdala signals likely reflect the activity of these two separate neuronal populations. The location of our main effects is consistent with basolateral and centromedial amygdala, where intermingled sequence value and sequence length neurons are found in monkeys [15, 16]. The coexistence of these signals in the same brain system—shown here for the first time in the human amygdala—might indicate local conversion from economic valuations to behavioral strategies [15, 16], potentially via competitive, inhibitory interactions among neighboring neurons. This process most likely involves frontal areas with known decision functions, which depend on interactions with amygdala [21, 22].

Compared to the monkey studies, the present human experiments provide several new insights. The currently reported planning signals in the human amygdala integrated multiple factors in the subjective valuation of saving plans, including interest rate and reward type. The present data also link amygdala planning activities to a sophisticated, perhaps human-specific form of economic behavior involving the formulation of bids in an auction-like (BDM) mechanism. Critically, we demonstrate that the amygdala's planning activity and amygdala-frontal connections partly explain inter-individual differences in saving behavior, which relates to key economic issues affecting individuals and societies [3]. Using whole-brain imaging allowed us to uncover functionally connected systems in frontal cortex beyond amygdala with previously unknown functions in economic saving. These frontal areas encode partly distinct planning components and thus represent interesting targets for future single-neuron recordings. Notably, the same amygdala-frontal circuits are implicated in deregulated reward expectation and affective disorders [50], which impact on the motivation to plan for future rewards and pursue distant goals. Our experimental approach to the neurobiology of economic saving could help understand dysfunctional planning and decision functions of amygdala-frontal circuits in such conditions.

Conclusions

Theories of planned behavior identify a two-stage process that distinguishes initial plan formation from subsequent execution, and a valuation component that directs behavioral strategies toward future rewards. The present data characterize the neural mechanisms underlying these processes during the formation and pursuit of simple economic saving strategies. Our findings suggest an extended view of the human amygdala that includes a planning function for future rewards embedded within prefrontal circuits with distinct planning and decision functions.

SUPPLEMENTAL INFORMATION

Supplemental Information includes five figures, five tables, and Supplemental Experimental Procedures and can be found with this article online at http://dx.doi.org/10.1016/j.cub.2016.09.016.

AUTHOR CONTRIBUTIONS

L.Z., F.G., and W.S. designed the research. L.Z performed experiments. L.Z. and F.G. analyzed the data. L.Z., F.G., and W.S. wrote the manuscript.

ACKNOWLEDGMENTS

The Local Research Ethics Committee of the Cambridgeshire Health Authority approved this study. All participants gave written consent before the experiment. We thank the Wellcome Trust (grant 095495 to W.S.), the European Research Council (grant 293594 to W.S.), and the US National Institutes of Health Conte Center at Caltech (grant P50MH094258 to W.S.) for financial support.

Received: June 17, 2016
Revised: August 18, 2016
Accepted: September 12, 2016
Published: October 20, 2016

REFERENCES

1. Benhabib, J., and Bisin, A. (2005). Modeling internal commitment mechanisms and self-control: A neuroeconomics approach to consumption–saving decisions. Games Econ. Behav. *52*, 460–492.
2. Prelec, D., and Loewenstein, G. (1998). The red and the black: mental accounting of savings and debt. Marketing Sci. *17*, 4–28.
3. Brown, A.L., Chua, Z.E., and Camerer, C.F. (2009). Learning and visceral temptation in dynamic saving experiments. Q. J. Econ. *124*, 197–231.
4. Miller, G.A., Galanter, E., and Pribram, K.H. (1960). Plans and the Structure of Behavior (Holt, Rinehart and Winston).
5. Sutton, R.S., and Barto, A.G. (1998). Reinforcement Learning (MIT Press).
6. Shallice, T., and Burgess, P.W. (1991). Deficits in strategy application following frontal lobe damage in man. Brain *114*, 727–741.
7. Tanji, J. (2001). Sequential organization of multiple movements: involvement of cortical motor areas. Annu. Rev. Neurosci. *24*, 631–651.
8. Wolbers, T., and Hegarty, M. (2010). What determines our navigational abilities? Trends Cogn. Sci. *14*, 138–146.
9. Sharot, T., Riccardi, A.M., Raio, C.M., and Phelps, E.A. (2007). Neural mechanisms mediating optimism bias. Nature *450*, 102–105.
10. Kable, J.W., and Glimcher, P.W. (2007). The neural correlates of subjective value during intertemporal choice. Nat. Neurosci. *10*, 1625–1633.
11. McClure, S.M., Ericson, K.M., Laibson, D.I., Loewenstein, G., and Cohen, J.D. (2007). Time discounting for primary rewards. J. Neurosci. *27*, 5796–5804.
12. Peters, J., and Büchel, C. (2010). Episodic future thinking reduces reward delay discounting through an enhancement of prefrontal-mediotemporal interactions. Neuron *66*, 138–148.
13. Doll, B.B., Duncan, K.D., Simon, D.A., Shohamy, D., and Daw, N.D. (2015). Model-based choices involve prospective neural activity. Nat. Neurosci. *18*, 767–772.
14. Wunderlich, K., Dayan, P., and Dolan, R.J. (2012). Mapping value based planning and extensively trained choice in the human brain. Nat. Neurosci. *15*, 786–791.
15. Grabenhorst, F., Hernádi, I., and Schultz, W. (2012). Prediction of economic choice by primate amygdala neurons. Proc. Natl. Acad. Sci. USA *109*, 18950–18955.
16. Hernádi, I., Grabenhorst, F., and Schultz, W. (2015). Planning activity for internally generated reward goals in monkey amygdala neurons. Nat. Neurosci. *18*, 461–469.
17. Paton, J.J., Belova, M.A., Morrison, S.E., and Salzman, C.D. (2006). The primate amygdala represents the positive and negative value of visual stimuli during learning. Nature *439*, 865–870.
18. Gottfried, J.A., O'Doherty, J., and Dolan, R.J. (2003). Encoding predictive reward value in human amygdala and orbitofrontal cortex. Science *301*, 1104–1107.
19. Grabenhorst, F., Rolls, E.T., Parris, B.A., and d'Souza, A.A. (2010). How the brain represents the reward value of fat in the mouth. Cereb. Cortex *20*, 1082–1091.
20. Schultz, W. (2015). Neuronal Reward and Decision Signals: From Theories to Data. Physiol. Rev. *95*, 853–951.
21. Murray, E.A., and Rudebeck, P.H. (2013). The drive to strive: goal generation based on current needs. Front. Neurosci. *7*, 112.
22. Rudebeck, P.H., Mitz, A.R., Chacko, R.V., and Murray, E.A. (2013). Effects of amygdala lesions on reward-value coding in orbital and medial prefrontal cortex. Neuron *80*, 1519–1531.
23. De Martino, B., Camerer, C.F., and Adolphs, R. (2010). Amygdala damage eliminates monetary loss aversion. Proc. Natl. Acad. Sci. USA *107*, 3788–3792.
24. De Martino, B., Kumaran, D., Seymour, B., and Dolan, R.J. (2006). Frames, biases, and rational decision-making in the human brain. Science *313*, 684–687.

25. Hampton, A.N., Adolphs, R., Tyszka, M.J., and O'Doherty, J.P. (2007). Contributions of the amygdala to reward expectancy and choice signals in human prefrontal cortex. Neuron *55*, 545–555.

26. Grabenhorst, F., Schulte, F.P., Maderwald, S., and Brand, M. (2013). Food labels promote healthy choices by a decision bias in the amygdala. Neuroimage *74*, 152–163.

27. Rutishauser, U., Ye, S., Koroma, M., Tudusciuc, O., Ross, I.B., Chung, J.M., and Mamelak, A.N. (2015). Representation of retrieval confidence by single neurons in the human medial temporal lobe. Nat. Neurosci. *18*, 1041–1050.

28. Bartra, O., McGuire, J.T., and Kable, J.W. (2013). The valuation system: a coordinate-based meta-analysis of BOLD fMRI experiments examining neural correlates of subjective value. Neuroimage *76*, 412–427.

29. Grafman, J. (2002). The structured event complex and the human prefrontal complex. In Principles of Frontal Lobe Function, D.T. Stuss, and R.T. Knight, eds. (Oxford University Press), pp. 292–310.

30. Hare, T.A., Schultz, W., Camerer, C.F., O'Doherty, J.P., and Rangel, A. (2011). Transformation of stimulus value signals into motor commands during simple choice. Proc. Natl. Acad. Sci. USA *108*, 18120–18125.

31. Kolling, N., Behrens, T.E.J., Mars, R.B., and Rushworth, M.F.S. (2012). Neural mechanisms of foraging. Science *336*, 95–98.

32. Croxson, P.L., Walton, M.E., O'Reilly, J.X., Behrens, T.E.J., and Rushworth, M.F.S. (2009). Effort-based cost-benefit valuation and the human brain. J. Neurosci. *29*, 4531–4541.

33. Small, D.M., Veldhuizen, M.G., Felsted, J., Mak, Y.E., and McGlone, F. (2008). Separable substrates for anticipatory and consummatory food chemosensation. Neuron *57*, 786–797.

34. Becker, G.M., DeGroot, M.H., and Marschak, J. (1964). Measuring utility by a single-response sequential method. Behav. Sci. *9*, 226–232.

35. Phelps, E.A., and LeDoux, J.E. (2005). Contributions of the amygdala to emotion processing: from animal models to human behavior. Neuron *48*, 175–187.

36. Rolls, E.T. (2015). Limbic systems for emotion and for memory, but no single limbic system. Cortex *62*, 119–157.

37. Janak, P.H., and Tye, K.M. (2015). From circuits to behaviour in the amygdala. Nature *517*, 284–292.

38. Phelps, E.A., Lempert, K.M., and Sokol-Hessner, P. (2014). Emotion and decision making: multiple modulatory neural circuits. Annu. Rev. Neurosci. *37*, 263–287.

39. Rutishauser, U., Mamelak, A.N., and Adolphs, R. (2015). The primate amygdala in social perception - insights from electrophysiological recordings and stimulation. Trends Neurosci. *38*, 295–306.

40. Seymour, B., and Dolan, R. (2008). Emotion, decision making, and the amygdala. Neuron *58*, 662–671.

41. Sakai, K., Rowe, J.B., and Passingham, R.E. (2002). Active maintenance in prefrontal area 46 creates distractor-resistant memory. Nat. Neurosci. *5*, 479–484.

42. Rowe, J.B., Owen, A.M., Johnsrude, I.S., and Passingham, R.E. (2001). Imaging the mental components of a planning task. Neuropsychologia *39*, 315–327.

43. Shidara, M., and Richmond, B.J. (2002). Anterior cingulate: single neuronal signals related to degree of reward expectancy. Science *296*, 1709–1711.

44. Bechara, A., Damasio, H., Damasio, A.R., and Lee, G.P. (1999). Different contributions of the human amygdala and ventromedial prefrontal cortex to decision-making. J. Neurosci. *19*, 5473–5481.

45. Hayden, B.Y., Pearson, J.M., and Platt, M.L. (2011). Neuronal basis of sequential foraging decisions in a patchy environment. Nat. Neurosci. *14*, 933–939.

46. Daw, N.D., O'Doherty, J.P., Dayan, P., Seymour, B., and Dolan, R.J. (2006). Cortical substrates for exploratory decisions in humans. Nature *441*, 876–879.

47. Chib, V.S., Rangel, A., Shimojo, S., and O'Doherty, J.P. (2009). Evidence for a common representation of decision values for dissimilar goods in human ventromedial prefrontal cortex. J. Neurosci. *29*, 12315–12320.

48. Grabenhorst, F., D'Souza, A.A., Parris, B.A., Rolls, E.T., and Passingham, R.E. (2010). A common neural scale for the subjective pleasantness of different primary rewards. Neuroimage *51*, 1265–1274.

49. Logothetis, N.K. (2008). What we can do and what we cannot do with fMRI. Nature *453*, 869–878.

50. Koob, G.F., and Volkow, N.D. (2010). Neurocircuitry of addiction. Neuropsychopharmacology *35*, 217–238.

Current Biology, Volume *26*

Supplemental Information

Neural Basis for Economic Saving Strategies in Human Amygdala-Prefrontal Reward Circuits

Leopold Zangemeister, Fabian Grabenhorst, and Wolfram Schultz

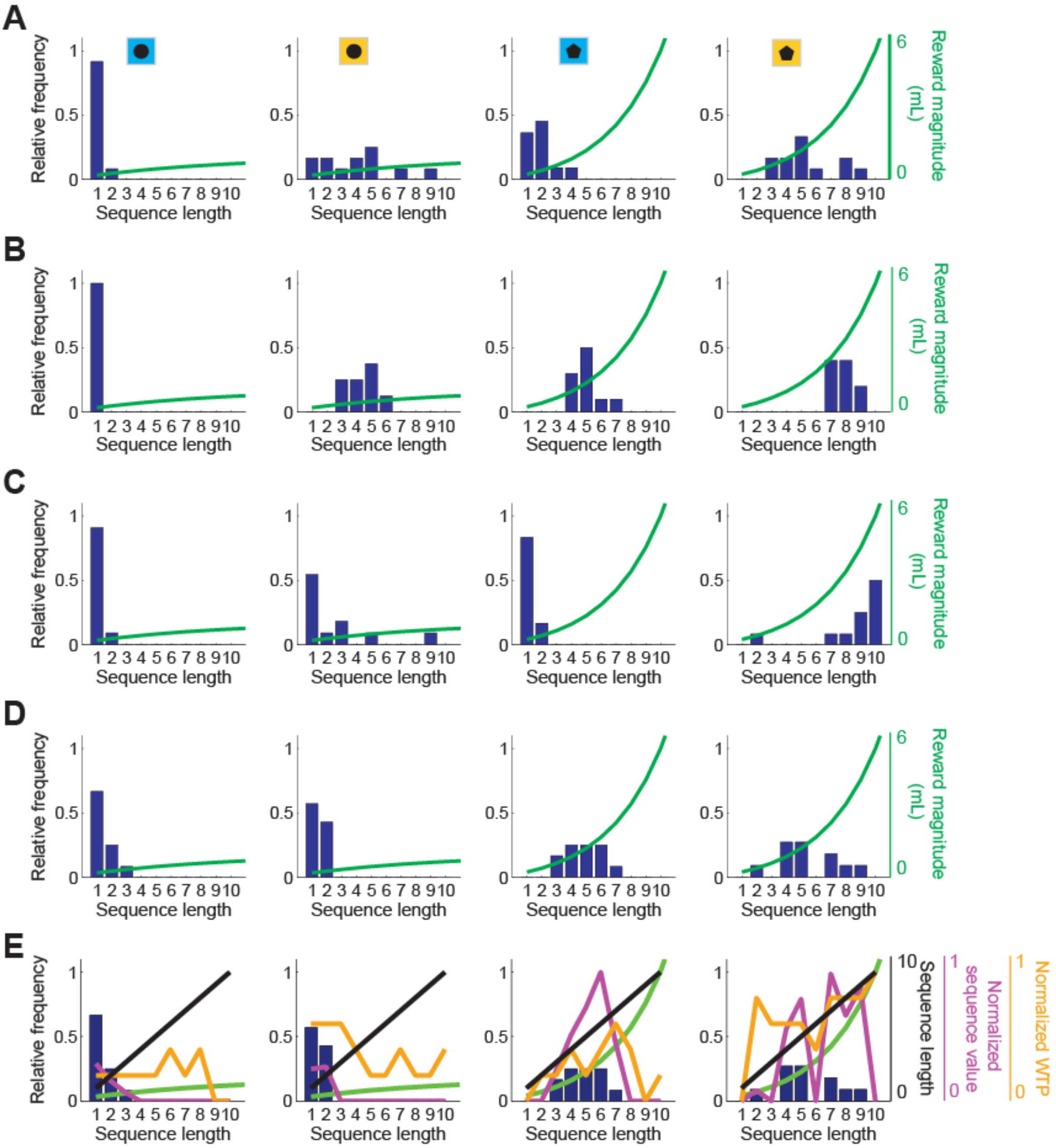

Figure S1 (related to Figure 1) | Choice frequencies in single subjects. A-D, Saving behavior in four representative subjects. Bars show relative frequencies with which the subject produced different choice sequences. Green curves show reward magnitude increases over sequential save choices. In each plot conditions are as follows (from left to right): Low fat, low interest; high fat, low interest; low fat, high interest; high fat, high interest. The figure illustrates variations in saving behavior both across subjects and experimental conditions. **E**, Same subject as in panel d. In addition to relative frequency and reward magnitude, the graphs show normalized sequence length (black) and normalized sequence value (magenta) regressors. These plots illustrate how sequence length and value could vary independently in our two-factorial design. Note how normalized sequence length increases linearly, while sequence value and WTP do not follow a linear pattern. Note also that sequence value estimates (magenta line) with a

value of zero result from subjects not choosing this particular sequence length during the experiment. Thus these zero values were not used in our main fMRI analysis to predict sequence value.

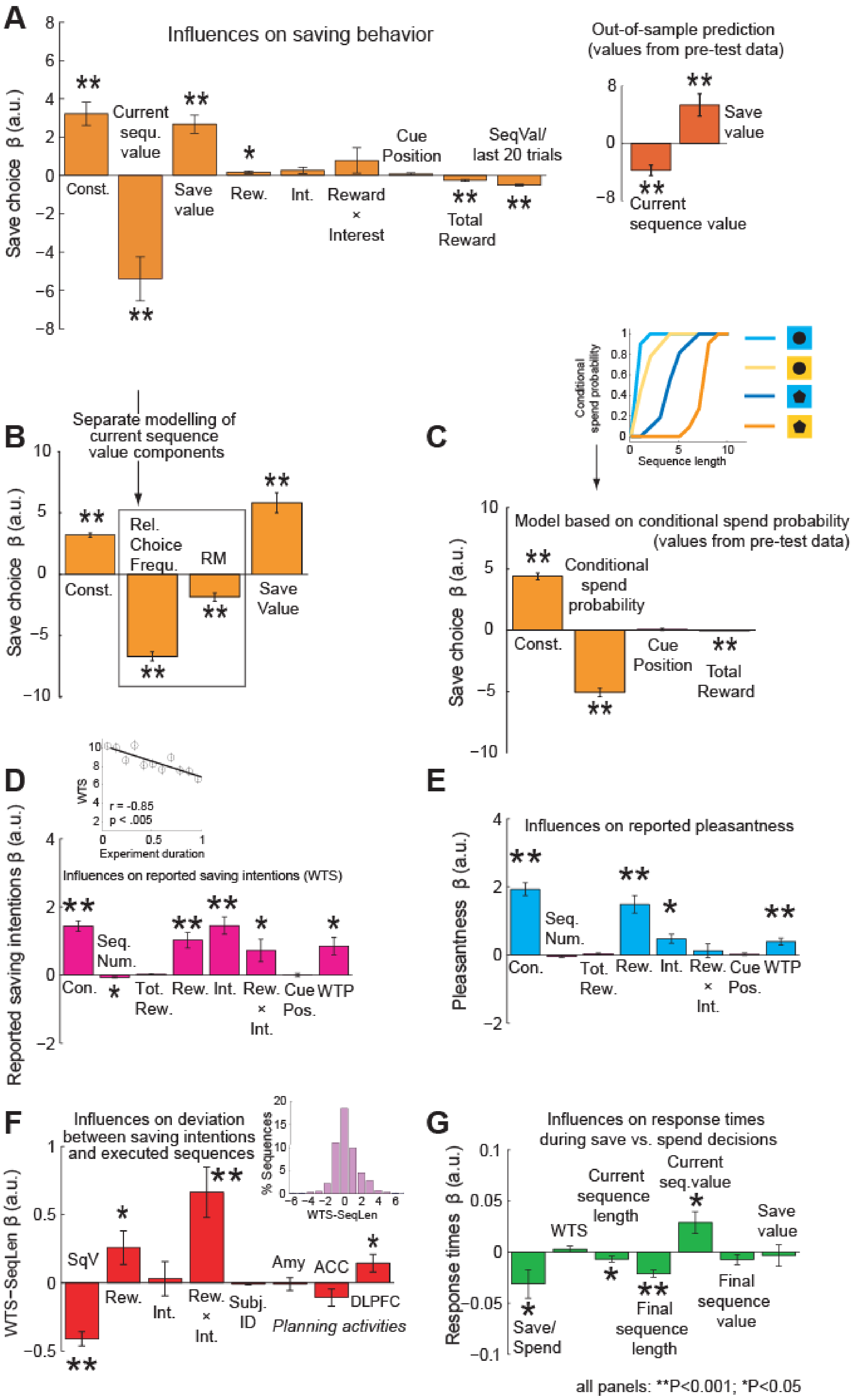

A
Influences on saving behavior
Save choice β (a.u.)
Const.
Current sequ. value
Save value
Rew.
Int.
Reward × Interest
Cue Position
Total Reward
SeqVal/ last 20 trials
Out-of-sample prediction (values from pre-test data)
Save value
Current sequence value
B
Separate modelling of current sequence value components
Save choice β (a.u.)
Const.
Rel. Choice Frequ.
RM
Save Value
C
Conditional spend probability
Sequence length
Model based on conditional spend probability (values from pre-test data)
Save choice β (a.u.)
Const.
Conditional spend probability
Cue Position
Total Reward
D
WTS
r = -0.85
p < .005
Experiment duration
Influences on reported saving intentions (WTS)
Reported saving intentions β (a.u.)
Con.
Seq. Num.
Tot. Rew.
Rew.
Int.
Rew. × Int.
Cue Pos.
WTP
E
Influences on reported pleasantness
Pleasantness β (a.u.)
Con.
Seq. Num.
Tot. Rew.
Rew.
Int.
Rew. × Int.
Cue Pos.
WTP
F
Influences on deviation between saving intentions and executed sequences
WTS−SeqLen β (a.u.)
% Sequences
WTS-SeqLen
SqV
Rew.
Int.
Rew. × Int.
Subj. ID
Amy
ACC
DLPFC
Planning activities
G
Influences on response times during save vs. spend decisions
Response times β (a.u.)
Save/ Spend
WTS
Current sequence length
Final sequence length
Current seq.value
Final sequence value
Save value
all panels: **P<0.001; *P<0.05

Figure S2 (related to Figures 1, 2, 4 and 5) | Behavioral regression analyses. A, Logistic regression of save-spend choices on subjective values (current sequence value, save value), reward type, interest rate, reward type × interest rate, left-right cue position, total reward (cumulative consumed reward in mL across sessions) and running average of sequence value across the last 20 trials. Shown are regression coefficients (± s.e.m.) obtained by fitting a logistic regression model to each subject's choices. Positive coefficients indicate a positive weight on save choice likelihood. Significance was tested by one-sample t-test on coefficients from all subjects (random-effects analysis). Current sequence value (t(23) = -4.72)) and save value (t(23) = 5.62) were the main weights on choices (reward: t(23) = 2.53; interest t(23) = 1.63; reward × interest (t(23) = 1.19; cue position (t(23) = 1.39; total reward (t(23) = -5.44); running average of sequence value across the last 20 trials (t(23) = -9.16)). Inset: value coefficients remained significant when values were derived from independent behavioral data of a pre-scanning session (out-of-sample prediction; P < 0.001; current sequence value: t(23) = -4.34; save value: t(23) = 3.83). Adding length of previous saving sequence did not affect results (last sequence length P > 0.05, t(23) = -0.7). **B**, Separate modeling of sequence value components. Logistic regression showing effect of relative choice frequency (t(23) = -18.34) and current-trial reward magnitude (t(23) = -5.49) **C,** Logistic regression showing effect of cumulative spend choice probability (t(23) = -14.37), cue position (t(23) = -0.13) and total accumulated reward (t(23) = -5.17) on choices. Cumulative choice probability was defined as the sum of relative choice frequencies up to the current trial derived from a separate behavioral session. **D**, Multiple linear regression on reported saving intentions (n = 22). Results show effects of sequence number (t(21) = -2.99), total reward (t(21) = 1.06), reward type (t(21) = 4.63), interest rate condition (t(21) = 5.69), their interaction (t(21) = 2.12), cue position (t(21) = 0.0002)) and willingness-to-pay for the current sequence (P < 0.05; t(21) = 3.35). Experiment progress had an effect but across subjects this was relatively small compared to other regressors of interest. Inset shows the distribution of deviations between reported saving intentions and chosen sequence lengths. **E**, Multiple linear regression (n = 22) showing effects of sequence number (t(21) = -1.47), total reward (t(21) = 1.59), reward type (t(21) = 5.77), interest rate condition (t(21) = 3.67), their interaction (t(21) = 0.61), cue position (t(21) = 0.56) and willingness-to-pay (t(21) = 3.94) on reported pleasantness of reward. **F**, Influences on deviation (WTS minus sequence length). Shown are regression coefficients (± s.e.m.) from a multiple linear regression analysis across subjects and trials (fixed effects). Sequence value (t(1010) = -7.81), reward type (t(1010) = 2.09), interaction of reward type and interest rate (t(1010) = 3.57) and DLPFC BOLD signal during planning (t(1010) = 2.23) each had a significant effect. **G**, Response time analysis. Shown are regression coefficients (± s.e.m.) from a multiple linear regression analysis across subjects and trials (fixed effects) on the response times. Response times were affected by the subjects' choice (save/spend dummy variable (0/1), t(4811) = -2.25), became shorter throughout a sequence (current sequence length, t(4811) = -2.34), were shorter in longer sequences (final sequence length, t(4811) = -6.04), were related to current sequence value (t(4811) = 2.67) but were not related to

reported saving intentions (WTS, $t(4811) = 0.96$) or final sequence value ($t(4811) = -1.54$) or save value ($t(4811) = -0.33$).

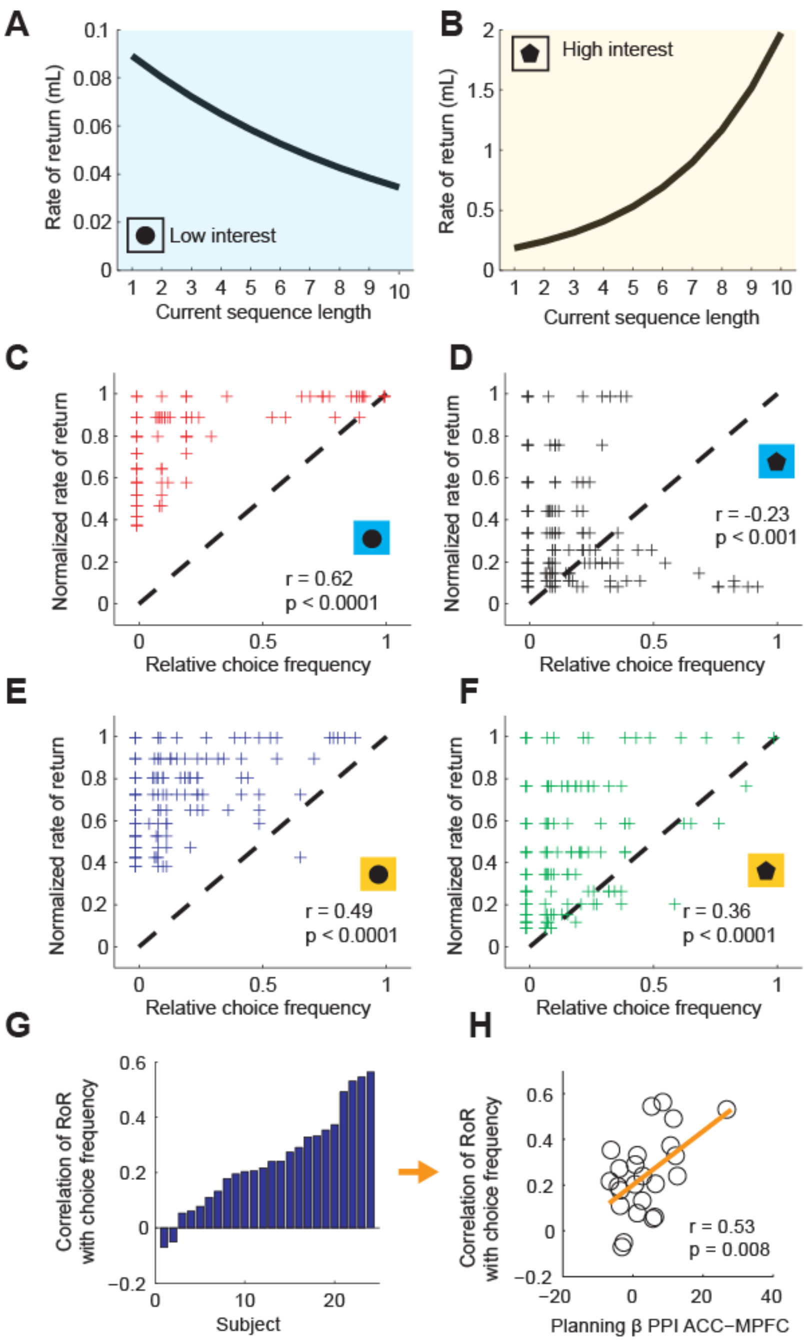

Figure S3 (related to Figures 1 and 4) | Relation of subjects' behavior to trial-by-trial rate of return. A-B, Diagrams showing the rate of return, defined as the additional reward (mL) to be gained by deciding to save in the current trial. **C-F,** scatter plots showing the relationship between relative choice frequency and rate of return. Subjects' observed relative choice frequencies were positively related to the rate of return in all conditions except the low fat, high interest condition. Here subjects showed shorter sequence lengths regardless of the positively developing rate of return. **G**, For each subject, we pooled the data across conditions and correlated rate of return with relative choice frequency. Shown is the distribution of correlation coefficients across subjects. **H**, across subjects, the matching of rate of return and choice frequency was related to connectivity strengths between ACC and MPFC during planning.

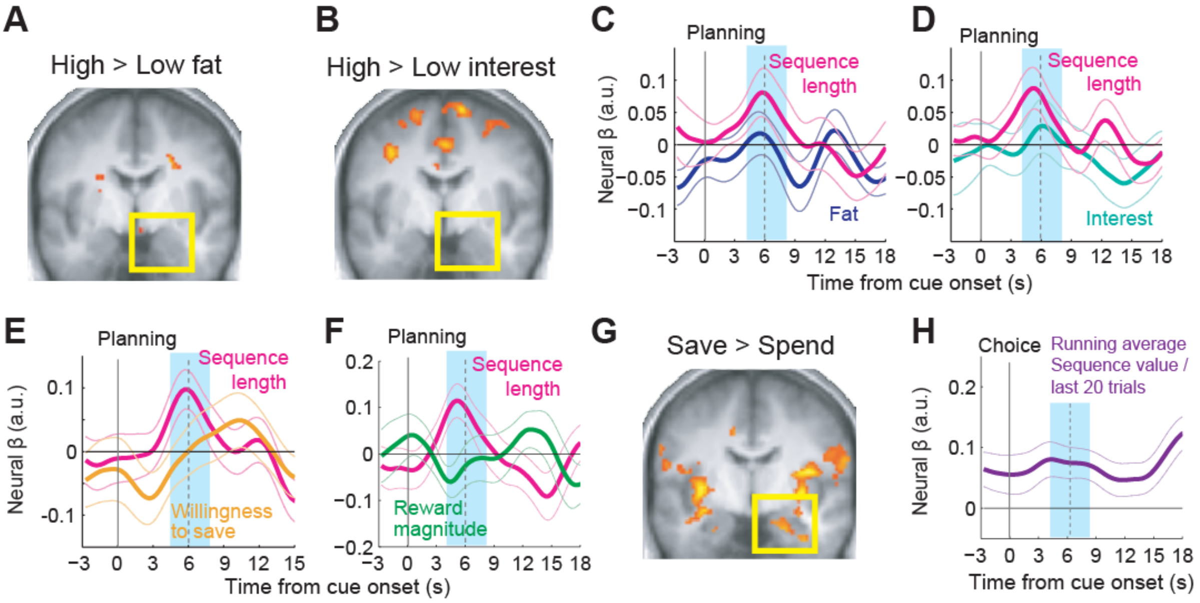

Figure S4 (related to Figures 2, 3 and 5) | Amygdala control analyses. A, Amygdala activity during the planning phase did not reflect simple cue differences between high and low fat reward conditions (non-significant effect in either direction, small volume correction, GLM5). We used the standard SPM8 settings by which regressors are orthogonalized in the order they are entered. Thus, the analysis shown here should have detected average cue effects for reward type and interest rate in amygdala if they existed. **B,** Amygdala activity during the planning phase did not reflect simple cue differences between high and low interest rate conditions (non-significant effect in either direction, small volume correction, GLM5). **C,** Region-of-interest analysis across planning phases in all trials. Regression of amygdala activity during the planning phase on sequence length and reward type. The GLM plotted here included regressors sequence length and an indicator function ("dummy variable") for reward type (1 = high fat; 0 = low fat). Sequence length regressor was orthogonalized with respect to reward type. Only sequence length explained significant variance ($P < 0.05$, random effects multiple linear regression; $t(23) = 2.12$). **D,** Region-of-interest analysis across planning phases in all trials. Regression of amygdala activity on sequence length and interest rate. The GLM plotted here included regressors sequence length and an indicator function ("dummy variable") for interest rate (1 = high interest; 0 = low interest). Sequence length regressor was orthogonalized with respect to interest rate. Only sequence length explained significant variance ($t(23) = 2.76$). **E,** Regression of amygdala activity on sequence length and willingness-to-save rating (saving intentions). Only sequence length explained significant variance ($t(23) = 3.12$). **F,** Region-of-interest analysis across planning phases in all trials. Regression of amygdala activity on sequence length and reward magnitude. Only sequence length explained significant variance ($t(23) = 3.13$). Further, including the sequence number as a proxy for duration for the experiment in a model along with sequence length to explain amygdala BOLD signal during planning had no effect on the correlation with BOLD and sequence length (sequence length still significant $P<0.05$, $t(23) = 2.85$). **G,** Stronger amygdala activity during save choices compared to spend

choices (cluster *P* values corrected for family-wise error across the whole-brain, $P < 0.05$; t-test $(23) = 3.93$; map thresholded at $P < 0.005$, uncorrected for display purposes, extent threshold ≥ 10 voxels). **H**, Region-of-interest analysis across choice phases in all trials. Regression of amygdala activity on running average of sequence value over the last 20 trials. Amygdala activity correlated with this variable during the choice phase ($P<0.05$, $t(23)= 2.96$). Similar effects were found in ACC ($t(23)=2.25$)) and DLPFC ($t(23)=2.19$)).

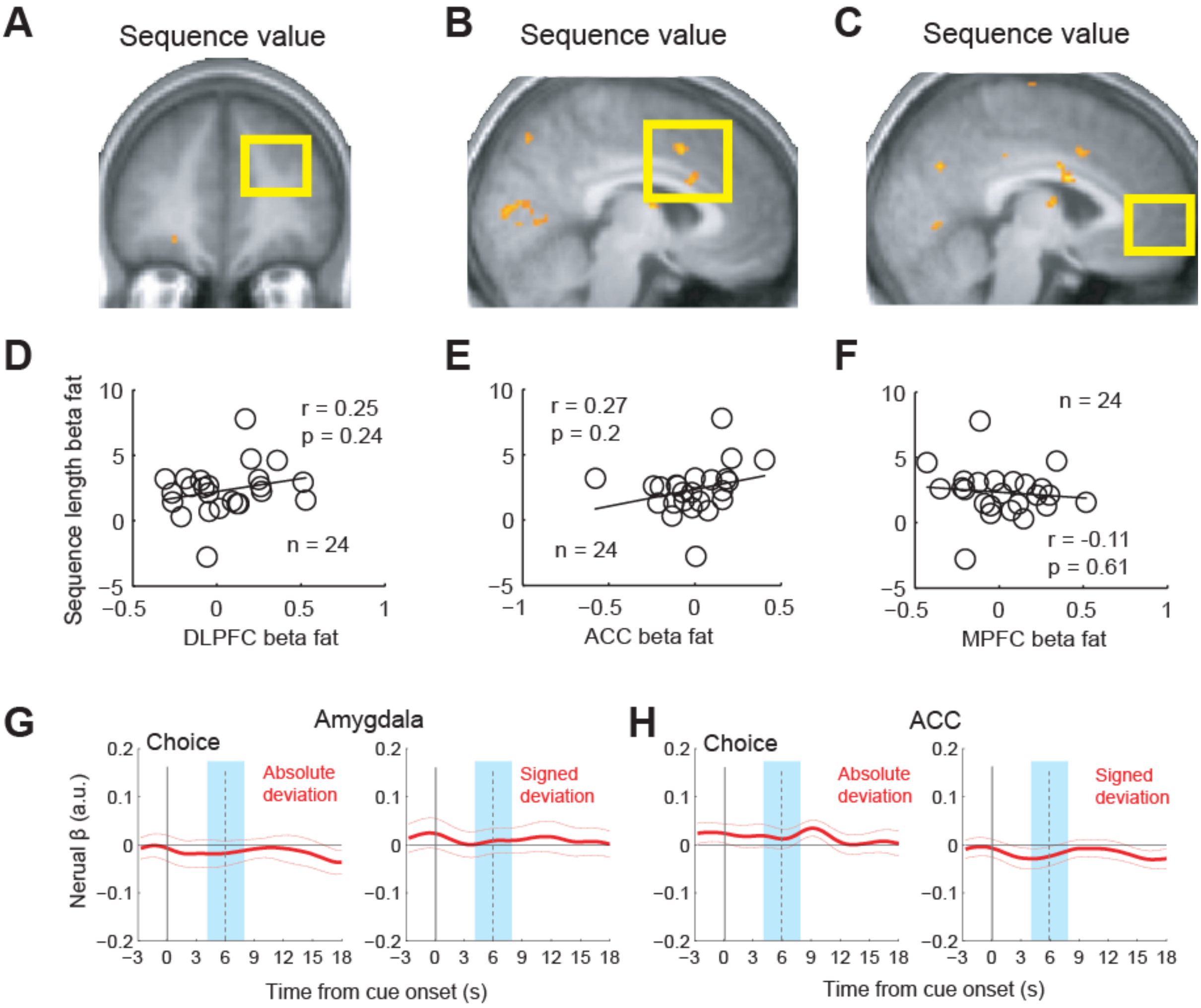

Figure S5 (related to Figures 2-5) | fMRI control analyses. A-C, Statistical maps for sequence value in the planning phase show no effect in DLPFC, ACC or MPFC (cluster *P* values corrected for family-wise error across the whole-brain, $P < 0.05$; map thresholded at $P < 0.005$, uncorrected for display purposes, extent threshold ≥ 10 voxels). No effects were present even at lower threshold of $P < 0.01$, uncorrected. **D-E,** Neurometric-psychometric comparison across subjects for DLPFC, ACC and MPFC. Behavioral and neural reward βs plotted for all subjects as shown for amygdala in Figure 3C. Behavioral sensitivity to reward was not significantly related to neural reward sensitivity in any of the three frontal areas. **G-H**, Region of interest analyses: Neither amygdala nor ACC activity reflected the absolute or signed difference between reported saving intentions and executed sequences during choice.

Table S1 (related to Figures 2 and 4). Whole-brain analysis (GLM 1) results related to contrast of planning phase vs. choice phase (cluster *P* values corrected for family-wise error across the whole-brain, $P < 0.05$; maps thresholded at $P < 0.005$, extent threshold ≥ 10 voxels).

Effect	Sign of correlation	Anatomical region	Hemisphere	MNI peak coordinates (x, y, z)	z-score
Planning phase > choice phase (GLM 1)	/	Amygdala	R	22, 0, -18	5.12
		DLPFC	R	34, 40, 32	5.99
		DLPFC	L	-32, 36, 36	6.27
		ACC	/	0, 15, 42	5.9
		Thalamus	L	-10, -18, 2	6.93
		Thalamus	R	8, -18, 2	6.54
		Cerebellum	L	-12, -62, -18	6.65
		Intraparietal sulcus	L	-38, -56, 42	6.5
		Postcentral gyrus	L	-58, -8, 22	6.48
		Insula	R	32, -10, 10	6.44
		Cerebellum	R	26, -50, -20	6.23
		Posterior cingulate cortex	L	-4, -20, 26	6.07

Table S2 (related to Figures 2 and 4). Whole-brain analysis (GLM1-2) results related to parametric variables during planning phase (cluster *P* values corrected for family-wise error across the whole-brain, $P < 0.05$; maps thresholded at $P < 0.005$, extent threshold ≥ 10 voxels). * $P < 0.05$, small volume corrected

Effect	Sign of correlation	Anatomical region	Hemisphere	MNI peak coordinates (x, y, z)	z-score
Sequence length during planning (GLM 1)	Positive	Amygdala*	R	22, -4, -20	3.18
		ACC	/	6, 20, 48	4.54
		DLPFC	R	38, 42, 26	3.76
		DLPFC	R	36, 4, 50	4.41
		Intraparietal sulcus	R	50, -42, 38	3.88
		Intraparietal sulcus	L	-26, -62, 50	3.78
		Posterior cingulate cortex	/	0, -22, 28	4
		Precuneus	R	12, -66, 32	4.2
		Paracentral lobe	L	-26, -22, 74	3.6
		Superior temporal gyrus	R	56, -26, -10	4.24
		Striate/Extrastriate cortex	L	-28, -84, 2	4.58
		Striate/Extrastriate cortex	R	28, -78, -8	4.47
Sequence value during planning (GLM 2)	Positive	Amygdala*	R	22, -8, -22	3.26
		Extrastriate cortex	R	22, -86, -10	3.82

Table S3 (related to Figure 5). Whole-brain analysis (GLM 6) results related to save vs. spend choice trials (cluster *P* values corrected for family-wise error across the whole-brain, $P < 0.05$; maps thresholded at $P < 0.005$, extent threshold ≥ 10 voxels). * Uncorrected at $P<0.005$.

**Effect**	**Sign of correlation**	**Anatomical region**	**Hemisphere**	**MNI peak coordinates (x, y, z)**	**z-score**
Save > spend (choice phase) (GLM 6)	/	Amygdala	R	22, -2, -20	3.41
		Cerebellum	L	-14, -38, -20	4.87
		Middle temporal gyrus	L	-32, -20, -6	4.35
		Temporoparietal junction	L	-60, -48, 22	4.28
Spend > save (choice phase) (GLM 6)	/	Striatum*	/	-6, 12, 8	4.22

Table S4 (related to Figure 5). Whole-brain analysis results related to parametric variables during choice phase (cluster *P* values corrected for family-wise error across the whole-brain, P < 0.05; maps thresholded at P < 0.005, extent threshold ≥ 10 voxels).

**Comparison**	**Correlation**	**Anatomical region**	**Hemisphere**	**Peak Coordinates (mm) (x, y, z)**	**z-score**
Sequence length during choice phase (GLM 1)	Positive	ACC	R	10, 12, 46	4.39
	Negative	MPFC	L	-8, 62, 24	4.36
Current sequence value during choice phase (GLM 3)	Negative	Amygdala	R	20, -2, -18	3.75
		MPFC	/	-4, 62, 10	5.08
		Insula/ parietal operculum	R	52, -8, 2	4.9
		Insula/ transverse temporal gyrus	L	-44, -18, 2	4.48
		Cerebellum	L	-8, -48, -16	4.17
Position during choice phase (‘current sequence length’) (GLM 4)	Positive	ACC	/	2, 20, 42	4.69
		Visual cortex	R	32, -84, 2	4.47
		Visual cortex	L	-20, -70, 34	4.44
		Middle frontal gyrus	R	52, 20, 36	4.03
	Negative	Insula	R	38, -8, 2	5.11
		Insula	L	-36, -6, -4	5.07
		MPFC	L	-8, 60, 12	4.45
		Cerebellum	L	-16, -58, -18	4.4

Table S5 (related to Figures 4-5). Whole-brain analysis results of PPI analyses (cluster *P* values corrected for family-wise error across the whole-brain, P < 0.05; maps thresholded at P < 0.005, extent threshold ≥ 10 voxels).

**Effect**	**Sign of correlation**	**Anatomical region**	**Hemisphere**	**MNI peak coordinates (x, y, z)**	**z-score**
PPI planning > choice, DLPFC seed (GLM PPI1)	/	ACC/MPFC	R	14, 46, 24	4.78
		Pregenual cingulate cortex	/	4, 50, -14	4.77
		MPFC	/	-6, 58, 34	4.54
		Striatum	L	-8, 18, 6	4.17
		Striatum	R	6, 16, 10	4.13
PPI choice > planning, DLPFC seed (GLM PPI1)	/	Intraparietal sulcus	R	40, -50, 42	6.21
		DLPFC	R	40, 40, 28	5.94
		Supplemental motor area	R	8, -2, 70	4.15
		Middle frontal gyrus	R	32, -6, 64	4.12
		ACC	/	8,2, 38	3.91
		Cerebellum	L	-30, -58, -28	3.89
		Middle temporal gyrus	L	-48, -60, -4	3.78
PPI choice > planning, amygdala seed (GLM PPI1)	/	Supplemental motor area	/	0, -2, 68	5.99
		ACC	/	-2, 12, 36	4.48
		Precentral gyrus	R	24, -12, 58	4.35
		Lateral temporal lobe	L	-38, -22, -16	4.57
		Amygdala	L	-18, -8, -20	4.54
		Striatum		0, -10, 12	4.27
		Middle temporal gyrus	L	-44, -58, 8	4.17
		Striate cortex	R	18, -92, -4	4.08
		Extrastriate cortex	R	30, -76, -16	4.06
		Pre-Supplemental motor area	R	16, 30, 48	4.06
		Pre-Supplemental motor area	L	-12, 26, 50	4.01
		Precentral gyrus	R	50, -10, 20	3.76
		Insula	R	34, -24, 12	3.66
PPI choice > planning, ACC seed (GLM PPI1)	/	ACC	/	2, 26, 44	5.79
		DLPFC	R	32, 48, 14	5.59
		Insular gyrus	R	50, 16, -8	5.52
		DLPFC	L	-28, 52, 12	4.92
PPI high > low fat (planning phase), amygdala seed (GLM PPI2)	/	ACC*	R	10, 28, 48	3.53
		Intraparietal sulcus	L	-30, -30, 66	3.4

PPI high > low interest (planning phase), ACC seed (GLM PPI3)		MPFC	R	18, 62, 12	3.5
PPI save > spend (choice phase), amygdala seed (GLM PPI4)	/	MPFC	R	18, 64, 26	3.4
		Ventromedial prefrontal cortex	/	0, 50, -8	3.19
		Intraparietal sulcus	R	22, -34, 76	4.06
		Cerebellum	/	-2, -62, -32	3.91
		Visual cortex	L	-28, -86, 2	3.81
		Visual cortex	R	28, -86, 0	3.57
		Precuneus	/	2, -54, 28	3.42
PPI save > spend (choice phase), ACC seed (GLM PPI4)	/	DLPFC	R	32, 52, 16	3.91
		Middle temporal gyrus	L	-62, -32, -8	4.84
		Postcentral gyrus	L	-14, -26, 66	4.7
		Medial/superior temporal gyrus	R	56, -22, -2	4.16
		ACC	R	8, 24, 52	4.1
		Ventrolateral prefrontal cortex	R	34, 54, 0	4.08
		Ventrolateral prefrontal cortex	L	-36, 48, 2	4.02
		Intraparietal sulcus	L	-50, -42, 46	3.93
		Insula	R	32, -14, 14	3.78
		Intraparietal sulcus	R	18, -50, 60	3.64

*uncorrected at P=0.005, extent threshold ≥ 10 voxels

SUPPLEMENTAL EXPERIMENTAL PROCEDURES

Participants

28 healthy individuals (age range: 18-33 years; 13 females) participated in the study. All participants had normal or corrected-to-normal vision. We had to exclude four participants from all analyses due to motion artefacts, giving a sample size of n = 24 for the main saving task fMRI experiment. For two participants, no data for the separate behavioral BDM task was available due to an error in the data being written into a file, giving a sample size of n = 22 for the BDM task. Participants were screened to ensure they were not lactose intolerant, generally liked dairy products, had normal appetite and were not actively trying to avoid fat or sugar in their diet. Female participants were not pregnant. None of the participants had a history of psychiatric illness. All participants were healthy according to self-report and had no recent history of medication apart from contraceptive. The Local Research Ethics Committee of the Cambridgeshire Health Authority approved the study. All participants gave written consent before the experiment.

Experimental design

Before the scanning session, each participant took part in a behavioral session on a separate occasion to learn the task. During this session, participants performed exactly the same task as in the scanner including delivery of the liquid rewards. Participants were asked to not eat or drink anything except water for at least 4 hours before attending each session. This was done to ensure that subjects were hungry and willing to perform a task towards gaining liquid food rewards. Stimuli were presented on a screen and responses were given by pressing specific keys on a keyboard (training session) or button box (scanning session). Stimulus presentation and operant reactions were controlled and recorded using Cogent (Wellcome Trust Centre for Neuroimaging, London, UK) in Matlab (Version R2013b, Mathworks, Natick, MA).

Economic saving task. Subjects performed choice sequences of self-defined lengths to save different liquid rewards. The rewards accumulated according to a given interest rate (see below for interest rate calculation). The design was a 2×2 factorial design with the factors reward type (high vs. low fat content) and interest rate (high vs. low interest). (We use the term ‘interest rate’ to provide an intuitive description of the variable that governed increases in reward across

save choices; this should not imply exact comparability with financial interest rates.) We used different reward types and interest rates to promote variation in subjects' saving behavior and to distinguish neural activity related to sequence length (which was a linear function of the number of save choices in a sequence) from activity related to sequence value (which depended on subjects' preferences for different reward types, reward amounts and their interaction). All frame durations in the task were jittered according to Poisson distributions with an additional jitter of ± 200 ms to avoid predictability and to increase fMRI image acquisition efficiency. On average, subjects performed 42.6 ± 1.4 saving sequences (mean ± s.e.m.) during the fMRI experiment, with an average 213 ± 2.8 save-spend choice trials. These numbers were calculated excluding error sequences and error trials (see below) which were also excluded from all fMRI analyses.

Planning phase. During the planning phase, pre-trained cues indicated current interest rate (high vs. low) and current reward type (high vs. low fat content). The order of conditions (combinations of reward type and interest rate) between sequences was pseudo-randomized to avoid predictability but ensuring even numbers of each condition. Following cue presentation for 2-3 s and an inter-stimulus interval (ISI) of 2-4 s, subjects rated their willingness-to-save on a visual analogue scale ranging from 0 (low willingness-to-save) to 10 (high willingness-to-save). The rating was followed by an ISI of 2-4 s before the start of the choice phase.

Choice phase. During the choice phase, subjects made trial-by-trial choices to save or spend the accumulated reward. Each choice trial began with the presentation of a question mark on the screen for 2-3 s which prompted subjects to consider their save vs. spend choice for that trial. Following a 2-4 s ISI, the save cue and spend cue appeared in left-right position and subjects indicated their choice with a button press. Left-right position of save and spend cues was randomized across trials. Button presses were self-timed with the requirement that subjects indicated their choice within 3 s. A save choice was followed by a 2-3 s feedback screen stating "Saved", without providing feedback about saved reward amounts. Thus, the subjects had to track internally the accumulated reward amounts over consecutive save choices. Consecutive choice trials were separated by an inter-trial interval of 2-6 s. In each saving sequence, subjects were required to make at least one save choice. Subjects could make up to ten consecutive save choices per sequence with a cycle time of approximately 13 s per trial. A failure to respond on any trial lead to an error feedback stating "Please repeat trial" and resulted in the repetition of the trial. Accumulated saved rewards were retained across error trials. If a subject made more than the allowed ten save choices in a sequence, they received the feedback "Saved too long",

which resulted in cancellation of the saving sequence. This error occurred only rarely during the scanning experiment (mean = 1.08 ± 0.2) as subjects were pre-trained.

Reward phase. The reward phase followed subjects' spend choice in each sequence. A spend choice was followed by a 2-3 s feedback screen stating "Receive X mL in 2 sec". The accumulated amount of liquid reward was then delivered via a custom-made system consisting of two peristaltic pumps (see below). After reward delivery subjects were instructed to keep the liquid in their mouth for 0.5 s before swallowing for 1.5 s. The reward delivery and swallowing periods were cued by a yellow and green fixation cross, respectively. Subjects then rated the experienced pleasantness of the liquid on a visual analogue scale ranging from of 0 (very unpleasant) to 10 (very pleasant). The general protocol and procedures for liquid reward delivery in the scanner were modelled on previous fMRI studies [S1, S2].

Liquid rewards. The two types of liquid rewards consisted of vanilla-flavoured dairy drinks that differed in fat content. The low fat version was composed of 400 mL of skimmed milk (0.2 % fat) and the high fat version consisted of 300 mL double cream and 100 mL full fat milk (34.5% fat). Total sugar content was equal for both drinks. 10 mL of vanilla extract was added to each drink. The stimuli were based on previous human fMRI studies in which it was found that these stimuli represent potent rewards that produce activation in major reward areas [S1, S2]. The drinks were mixed in a beaker and kept cool using customized can coolers.

Reward delivery. The accumulated amount of liquid reward was delivered via a custom made system consisting of two peristaltic pumps (Experimental Psychology Workshop, University of Cambridge). Pumps were placed outside the scanner room in the control room. They were connected to a computer using an external National Instruments card (NI-USB-6009, National Instruments, Austin, Texas) and controlled via the Matlab Data Acquisition Toolbox. Participants received the liquid through a custom-made mouthpiece which were connected to silicone tubes of about 10 m length explicitly suitable for foodstuff (VWR International Ltd, UK).

Interest rate calculation. Growth in reward over consecutive save choices was calculated according to

$$x_n = b\sum_{i=0}^{n-1} q^i$$

with x_n as reward magnitude on trial n, b as the base rate of reward magnitude, and q as the interest rate [S3, S4]. The interest rate was either high ($q = 1.3$) or low ($q = 0.9$), resulting in a quasi-hyperbolic growth profile for the low q and quasi-exponential growth profile for the high q (green curves in Figure 1d). Base rate was set to $b = 0.11$. Interest rates and reward magnitudes were chosen based on behavioral pre-testing to ensure that subjects could discriminate the different reward magnitudes and were still able to drink the highest reward magnitude (6.2 mL) in the scanner. The following provides an example of how reward magnitudes were calculated. With a base rate of b = 0.11 and an interest rate of q = 1.3, on the first trial of the choice sequence the reward magnitude (RM) would correspond to RM = 0.11 × (1 + 1.3) = 0.25 ml. On the second trial, with two successive save choices, RM = 0.11 × (1 + 1.3 + 1.3^2) = 0.44 ml. On the third trial, with three successive save choices, RM = 0.11 × (1 + 1.3 + 1.3^2 + 1.3^3) = 0.68 ml. The interest rate calculation adopted for this experiment does not exactly match calculations commonly employed in financial theory. The definition described above was used in order to yield a decreasing marginal increase of reward for the low interest condition (see Figure 1D left most panel and third panel from the left).

Auction-like economic valuation task. Volunteers bid for different options in an adaptation of the Becker-DeGroot-Marschak [S5] auction-like task. Options mimicked the available combinations of sequence length and reward magnitude for each experimental condition (defined by combinations of reward type and interest rate) of the saving task. Specifically, each option consisted of a combination of reward magnitude (mL), reward type (high/low fat), and sequence length. There were 40 options in total, one for every possible sequence length in each of the four conditions. An example option would be “save 7 times to receive 2.6 mL of the high fat reward”. Information about the required number of save choices and the available reward magnitudes were provided in text form, whereas information about reward type was shown in the same way as in the main saving task, i.e. using a colored cue (Figure 1C). After viewing the current option, the phrase “bid?” appeared below the option, followed by a response by the subject in the range of 1 (low) to 5 (high) on a keyboard. Subjects were informed that not all auctions and related saving sequences would be implemented but that a small number of auctions would be selected randomly by the computer. Three second-price-auctions were

randomly implemented, one each between trials 5-10, 15-20 and 25-30. For each auction one randomly chosen bid placed by the subject was compared to a randomly generated number between 1 and 5. If the subject's bid was higher or equal to the randomly generated number, the subject "won" the auction. Winning the auction resulted in guided performance of the sequence that the subject had bid for. Each subject started with a certain number of points as their endowment, the remainder of which could be converted into drink after the task at an exchange rate of 1 point to 0.5 mL of drink. Volunteers were carefully instructed about the rules of the task to yield true valuations of each option. Post-instruction questionnaires confirmed that subjects understood the task rules and the different choice options. Subjects indicated that they found the description of the task in terms of save-spend decisions intuitive.

Behavioral data analysis

Saving index. To quantify differences in saving behavior between subjects and conditions, we calculated a saving index as follows (Figure 1E). Within each subject and condition, we determined the frequency of observing a saving sequence of a specific length relative to all possible saving lengths (Figure 1D). These relative frequencies summed to 1.0 across sequence lengths within a given condition. We then weighted (multiplied) these relative frequencies with their associated sequence lengths, thereby giving higher weight to higher sequence lengths. We then calculated the mean over these weighted sequence lengths for a given condition (defined by combination of reward type and interest rate). Thus,

$$Saving\ index_q = \frac{1}{n}\sum_{i=1}^{n} P_{i,q} SL_i,$$

with $Saving\ index_q$ as the saving index for a given condition q (defined by a combination of reward type and interest rate), n as the maximal sequence length (n = 10 in all conditions), $P_{i,q}$ as the mean relative frequency of observing a saving sequence of length i in condition q, and SL_i as the number of successive save choices required to obtain sequence length i.

Subjective values. As economic choices critically depend on the subjective values individuals derive from choice options, we estimated subjective values associated with specific saving sequences, following our previous approach from monkey experiments [S3, S4]. These

subjective values depended on final reward amounts and current reward type but also on expenditure related to sequence length. As higher reward amounts required longer sequences (determined by current interest rate), the value of the sequence was compromised by temporal delay and physical effort. To capture these influences on value in a direct manner, we followed the general notion of standard economic choice theory and estimated subjective values from observed behavioral choices.

We estimated the subjective value of different saving sequences by calculating the relative frequency with which each sequence length was chosen within a given condition. We then multiplied this frequency with the objective reward magnitude associated with the sequence length (Figure 1D, green curves). As identical sequence lengths were associated with different reward magnitude for different interest conditions, we multiplied these relative choice probabilities with objective reward magnitudes to account for magnitude differences between interest rates. This definition follows general economic approaches whereby reward magnitudes are weighted by their probability of occurrence. Thus, the subjective value for spending at any position i in the choice sequence was defined as

$$Sequence\ value_i = P_i \times RM_i$$

with P_i as the relative frequency of observing a spend choice at a given point i in a saving sequence (defined by the number of consecutive save choices) and with RM_i as the reward magnitude (in mL) resulting from spending on that trial. The sequence value actually realized in a specific saving sequence (which we call 'sequence value' in the paper) constituted the subjective value of that sequence, which was our main value regressor for neural activity in the planning phase (Figure 2C, D). We defined the sequence value as choice probability weighted by reward magnitude in order to account for value differences between interest rates conditions: for high interest rates, a given sequence length was associated with higher reward magnitude (compared to low the interest rate) which likely resulted in higher subjective value. A supplemental logistic regression indicated that choice frequency and reward magnitude accounted for separate variance in subjects' trial-by-trial choices (Figure S2B), consistent with previous results in monkeys [S3]. The sequence value associated with a given trial in a sequence, irrespective of whether the subject chose to spend on that trial ('current sequence value'), was used for logistic regression of trial-by-trial choices on values (Figure 1F, Figure S2) and constituted our main value regressor for neural activity in the choice phase (Figure 5A,

B, G, H). For comparisons across subjects, sequence value was normalized to the maximum value in each subject. Out-of-sample prediction confirmed that subjective values elicited in the first session (day 1, behavior only) predicted choices in the scanning session (day 2) well (Figure S2A, inset).

To model trial-by-trial save-spend choices, we defined the value of a save choice at a given position in a saving sequence ('save value') as the average sequence value associated with all potential future trials of that sequence. Thus, the subjective value for saving at a given point n in a sequence was

$$Save\ value_n = \frac{1}{m-n} \sum_{i=n+1}^{m} Sequence\ value_i,$$

with m defining the upper limit of the saving sequence (given by the maximal observed sequence length for the subject and condition). Thus, 'current sequence value' and 'save value' reflected trial-by-trial valuations, whereas 'sequence value' constituted the value of the finally chosen sequence.

The adequacy of these value definitions for modelling saving behavior was demonstrated previously in monkey experiments [S3, S4], and was confirmed in the present human study by a logistic regression of save-spend choices on values (Figure 1F, Figure S2), by significant correlation of subjective values with stated saving intentions (R = 0.42, P < 0.001), and by correlation of subjective values with subjects' bids in the BDM task (R = 0.39, P < 0.001).

Linear and logistic regression analysis of behavior. We used the following multiple regression analyses to examine influences on subjects' saving behavior. All regressions were performed at the random-effects level (i.e. regression coefficients were estimated separately for each subject and then entered into one-sample t-tests at the group level). To assess the influence of the objective factors reward type and interest rate and their interaction on subjects' saving behavior, we performed the following linear regression:

$$Sequence\ length = \beta_0 + \beta_1 Reward + \beta_2 Interest + \beta_3 Reward\ \times Interest + \varepsilon$$

with $Sequence\ length$ as the observed sequence length, $Reward$ as the current reward type

(dummy variable for high vs. low fat content, with 1 indicating high fat and 0 indicating low fat), $Interest$ as the current interest rate (dummy variable for high vs. low interest rate with 1 indicating high interest and 0 indicating low interest), $Reward \times Interest$ as interaction term, β_0 as constant term, β_1 to β_3 as the corresponding slope parameter estimates, and ε as residual.

In a second regression we tested whether there was an effect of the length of the last sequence on choice behavior by adding the factor $Last\ sequence\ length$ to the model described above:

$$Sequence\ length = \beta_0 + \beta_1 Reward + \beta_2 Interest + \beta_3 Reward \times Interest + \beta_4 Last\ sequence\ length + \varepsilon$$

To model trial-by-trial save-spend choices, we used the subjective values defined above ('current sequence value' and 'save value') as explanatory variables in a logistic regression model (Figure 1F, Figure S2A):

$$y = \beta_0 + \beta_1 Sequence\ value + \beta_2 Save\ value + \beta_3 Reward + \beta_4 Interest + \beta_5 Reward \times Interest + \beta_6 Cue\ position + \beta_7 Total\ reward + \beta_8 SeqVal\ running\ average + \varepsilon$$

with y as trial-by-trial save-spend choice (0 indicating spend choice, 1 indicating save choice), $Sequence\ value$ as current sequence value, $Save\ value$ as save value, $Cue\ position$ as the left-right position of the save cue (0 indicating left, 1 indicating right) to model potential side biases, $Total\ reward$ as a running index of consumed liquid over the whole experiment to model potential satiation effects, $SeqVal\ running\ average$ as the average obtained sequence value over the last 20 trials, β_0 as constant term, β_1 to β_8 as the corresponding slope parameter estimates, and ε as residual. We also performed an out-of-sample prediction using the behavioral data from the first testing session (day 1) to derive subjective values and predict choices in the subsequently performed scanning session (Figure S2A, inset).

To separately model the sequence value components we performed the following logistic regression model:

$$y = \beta_0 + \beta_1 Relative\ Choice\ Frequency + \beta_2 Reward\ Magnitude + \beta_3 Save\ value + \varepsilon$$

with y as trial-by-trial save-spend choice (0 indicating spend choice, 1 indicating save choice), $Relative\ Choice\ Frequency$ as relative choice frequency of the current sequence length, $Reward\ Magnitude$ as reward magnitude available on the current trial and other variables defined as above. The results are shown in Figure S2B.

In a further analysis, we modeled choices in terms of the observed cumulative probability to spend on a given trial, which we derived from separate behavioral data collected during the first testing session. The regression was of the following form:

$$y = \beta_0 + \beta_1 P(Spend) + \beta_2 Cue\ position + \beta_3 Total\ reward + \varepsilon$$

with y as trial-by-trial save-spend choice (0 indicating spend choice, 1 indicating save choice), $P(Spend)$ as cumulative spend probability over consecutive save trials derived from separate data and other variables as defined above. The results are shown in Figure S2C.

To analyse the influences on reported saving intentions and reported pleasantness we performed two separate multiple linear regression analyses of the following form:

$$y = \beta_0 + \beta_1 Sequence\ number + \beta_2 Total\ Reward + \beta_3 Reward + \beta_3 Interest + \beta_5 Reward \times Interest + \beta_6 Cue\ position + \varepsilon$$

with y being either willingness-to-save or subjective pleasantness, $Sequence\ number$ as running index of sequences performed over the whole scanning experiment, i.e. across all three runs (with the first performed sequence in the experiment taking the value of 1) and other variables as defined above. The resulting data are shown in Figure S2D-E.

To examine deviations between sequence length (observed behavior) and willingness-to-save (reported saving intentions), we performed the following multiple linear regression analysis:

$$y = \beta_0 + \beta_1 Sequence\ Value + \beta_2 Reward + \beta_3 Interest + \beta_3 Reward \times Interest + \beta_5 Subject\ ID + \beta_6 Amygdala\ planning\ activity + \beta_7 ACC\ planning\ activity + \beta_8 DLPFC\ planning\ activity + \varepsilon$$

with y being the deviation (i.e. signed difference) between saving intentions and sequence length (willingness-to-save – sequence length), , $Amygdala\ planning\ activity$ as the peak

BOLD signal in amygdala during the planning phase, $ACC\ planning\ activity$ as the peak BOLD signal in ACC during the planning phase, $DLPFC\ planning\ activity$ as the peak BOLD signal in DLPFC during the planning phase, and other variables as defined above. The resulting data are shown in Figure S2F.

Response time analysis. To analyse the influences on response times we performed a multiple linear regression analysis of the following form:

$$y = \beta_0 + \beta_1 Save\ vs.spend + \beta_2 WTS + \beta_3 Current\ sequence\ length \\ + \beta_4 Final\ sequence\ length + \beta_5 Current\ sequence\ value \\ + \beta_6 Final\ sequence\ value + \beta_7 Save\ value + \varepsilon$$

with y being the response time for the respective trial, $Save\ vs.spend$ as a dummy variable for save (1) or spend (0), WTS as reported saving intentions, $Current\ sequence\ length$ as the current sequence length, i.e. the position within the current sequence, $Final\ sequence\ length$ as the final sequence length of the current sequence, $Current\ sequence\ value$ as the sequence value available if the subject were to spend immediately, $Final\ sequence\ value$ as the sequence value obtained in that sequence by the subject, $Save\ value$ as the save value for the current trial within the sequence, β_0 as constant term, β_1 to β_7 as the corresponding slope parameter estimates, and ε as residual. The resulting data are shown in Figure S2G. To assess whether response times systematically decreased or increased across the scanning session we regressed response times on the current trial number across the whole scanning experiment. The data are described in the Results section 'Saving behavior and subjective value model'. Since subjects often deviated from their reported willingness-to-save, we were interest in investigating the hypothesis that response times are faster in spend trials in which subjects spent earlier than indicated by their willingness-to-save ('premature spend trial') compared to when they meet their reported willingness-to-save. We calculated the mean response time for each of these two trial types for each subject and in a second step entered these means into a second-level t-test across subjects. The results are described the Results section 'Saving behavior and subjective value model'.

Relationship of relative choice frequency and rate of reward return. We tested whether subjects' observed behavior (relative choice frequency) was related to the rate of reward return, defined as the additional reward magnitude to be gained (mL) by choosing to save in the current

trial. To this end we pooled the relative choice frequency of all subjects for each condition and regressed this on the rate of return for the corresponding trial. Perfect matching of choice frequency to rate of return would result in a positive linear relationship between the two variables. The results are shown in Figure S3 and mentioned in the Results section 'Saving behavior and subjective value model'.

fMRI data acquisition

We acquired echo T2*-weighted echo-planar images (EPIs) with blood-oxygen-level-dependent (BOLD) contrast using a Siemens 3T Trio Scanner at the Wolfson Brain Imaging Centre, Cambridge, UK. Data were acquired with in plane resolution 3 × 3 × 2 mm, 2 mm slice thickness, 56 slices, repetition time (TR) = 3 s, echo time (TE) = 30 ms, flip angle = 90° and field of view = 192 mm. Between 401 and 470 volumes were acquired in three separate runs for each participant, along with 4 "dummy" volumes before each scanning run. The acquisition plane was tilted by -30 degrees with respect to the anterior commissure–posterior-commissure axis and a z-shim gradient pre-pulse was applied to minimize signal dropout in inferior frontal and medial temporal lobe areas [S6]. High-resolution T1 structural scans were acquired using an MPRAGE sequence and co-registered to enable group level anatomical localization with the following sequence parameters: 1 × 1 × 1 mm^3 voxel resolution, 1 mm slice thickness, TR = 2.3 s, TE = 2.98 ms, inversion time 900 ms, flip angle = 9°.

fMRI data analysis

We performed the fMRI data analysis using statistical parametric mapping (SPM8; Wellcome Trust Centre for Neuroimaging, London). Preprocessing included realignment of functional data including motion correction, normalization to the Montreal Neurological Institute (MNI) coordinate system, and smoothing with a Gaussian kernel with full width at half maximum (FWHM) of 6 mm. A high-pass temporal filter with a cut-off period of 128 s was applied. General linear models (GLMs) assuming first-order autoregression were applied to the time course of activation in which event onsets were modelled as single impulse response functions convolved with the canonical hemodynamic response function. Time derivatives were included in the basis functions set. Linear contrasts of parameter estimates were defined to test specific effects in each individual dataset. Voxel values for each contrast resulted in a statistical parametric map of the corresponding *t* statistic. In the second (group random-effects) stage,

subject-specific linear contrasts of these parameter estimates were entered into one-sample t-tests, as described below, resulting in group-level statistical parametric maps. We estimated the following GLMs to test specific hypotheses:

GLM 1. This GLM served three purposes: (1) to identify brain areas more strongly activated in the planning phase compared to the choice phase (Figures 2A, 4A), (2) to search for regions correlating with the length of the forthcoming sequence ('sequence length') during the planning phase (Figures 2B, 4B), and (3) to search for regions correlating with the final length of the current choice sequence during the choice phase. For each subject we estimated a GLM with the following regressors of interest: (R1) an indicator function for the choice phase, i.e. the times when subjects were presented with the question mark cue prompting them to consider their save-spend decision for the current trial; (R2) R1 modulated by the final sequence length of the current choice sequence; (R3) an indicator function for the action phase, i.e. the times when the save and spend cue were presented and the subject could enter their choice using the button box; (R4) R3 modulated by an indicator function indicating whether the subject chose the cue presented on the left or on the right; (R5) an indicator function for the planning phase, i.e. the times when cues indicating interest rate and reward type were shown; (R6) R5 modulated by the length of the forthcoming choice sequence ('sequence length'); (R7) an indicator function for the willingness-to-save rating phase, i.e. the times when subjects indicated their willingness-to-save on a visual analogue scale from 0 (low) to 10 (high); (R8) an indicator function for the reward delivery period, i.e. the times when reward was delivered into the subject's mouth; (R9) R8 modulated by the reward magnitude (in mL); (R10) an indicator function for the pleasantness-rating phase, i.e. the times when the subjects indicated the pleasantness of the received reward; (R11-R17) the motion parameters resulting from the realignment pre-processing step as covariates of no interest; (R18-R20) three session constants.

GLM 2. This GLM identified regions associated with sequence value (Figure 2C, Figure S5A-C). It included the following regressors: (R1) an indicator function for the choice phase; (R2) R1 modulated by the sequence value (i.e. the final chosen sequence value) of the current choice sequence. (R3) an indicator function for the action phase; (R4) R3 modulated by an indicator function indicating whether the subject chose the cue presented on the left or on the right; (R5) an indicator function for the planning phase; (R6) R5 modulated by sequence value; (R7) an indicator function for the willingness-to-save rating phase; (R8) an indicator function for the reward delivery period; (R9) R8 modulated by the pleasantness rating; (R10) an indicator

function during the pleasantness-rating phase. The remaining details were the same as in GLM1.

GLM 3. This GLM identified regions associated with the trial-by-trial evolving ‘current sequence value’ (Figure 5A, G). It included the following regressors: (R1) an indicator function for the choice phase; (R2) R1 modulated by current sequence value; (R3) R1 modulated by save value; (R4) R1 modulated by sequence value (i.e. the final chosen sequence value); (R5) An indicator function for the action phase; (R6) R5 modulated by an indicator function indicating whether the subject chose the cue presented on the left or on the right; (R7) an indicator function for the planning phase; (R8) R7 modulated by sequence value; (R9) an indicator function for the willingness-to-save rating phase; (R10) an indicator function for the delivery period; (R12) an indicator function during the pleasantness-rating phase. The remaining details were the same as in GLM1.

GLM 4. This GLM identified areas in which activity correlates with the current sequence length during the choice phase (Figure 5E). It included the following regressors: (R1) an indicator function for the choice phase; (R2) R1 modulated by the current sequence length of the on-going choice sequence; (R3) R1 modulated by the final sequence length of the current choice sequence; (R4) an indicator function for the action phase; (R5) R4 modulated by an indicator function indicating whether the subject chose the cue presented on the left or on the right; (R6) an indicator function for the planning phase; (R7) R6 modulated by sequence length; (R8) an indicator function for the willingness-to-save rating phase; (R9) an indicator function for the delivery period; (R10) R9 modulated by the pleasantness rating; (R11) an indicator function during the pleasantness-rating phase. The remaining details were the same as in GLM1.

GLM 5. This GLM served to test whether parametric effects during the planning phase could be explained by the objective factors fat and interest (Figure S4A,B). The model contained the following regressors: (R1) an indicator function for the choice phase; (R2) R1 modulated by current sequence length; (R3) an indicator function for the action phase; (R4) R3 modulated by an indicator function indicating whether the subject chose the cue presented on the left or on the right; (R5) an indicator function for the planning phase in the low interest, low fat condition; (R6) R5 modulated by sequence length; (R7) an indicator function for the planning phase in the high interest, low fat condition; (R8) R7 modulated by sequence length; (R9) an indicator function for the planning phase in the low interest, high fat condition; (R10) R9 modulated by

sequence length; (R11) an indicator function for the planning phase in the high interest, high fat condition; (R12) R11 modulated by sequence length. The remaining details are the same as for GLM1.

GLM 6. This GLM served to contrast trials in which subjects chose to save with those in which they chose to spend (i.e. consume) (Figure S4G). It included the following regressors: (R1) an indicator function for the choice phase in trials in which the subject chose to save; (R2) an indicator function for the choice phase in trials in which the subject chose to spend; R3 to R10 were the same as in GLM 1. The remaining details are the same as for GLM1.

Functional connectivity analysis. We assessed functional connectivity using the psychophysiological-interaction (PPI) approach [S7, S8]. For each subject we first extracted eigenvariates for a 6 × 6 × 6 voxel cluster around a seed voxel based on the peak voxels identified by the correlation with sequence length during the planning phase (the main planning variable in the economic saving task). The peak voxel used for each subject was determined using a leave-one-out procedure by re-estimating our second level analysis 23 times, each time leaving out one subject. Starting at the respective peak voxel for correlation with sequence length we selected the nearest peak in these cross-validation analyses. Time courses were de-convolved with the canonical hemodynamic response function (HRF) to construct a time series of neural activity in the region of interest. We estimated the following PPI GLMs to test specific hypotheses.

PPI 1. This GLM tested for differential coupling between brain areas as a function of task phase (planning vs. choice phase). The results are shown in Figures 4E and 5I. The model contained the following regressors: (R1) a psychophysiological interaction regressor between the time series of activity in a seed brain area, extracted as just described, and a contrast between planning phase vs. choice phase; (R2) the time series of activity in a seed brain area, extracted as just described; (R3) a contrast between planning phase vs. choice phase; (R4-R9) the motion parameters resulting from the realignment pre-processing step as covariates of no interest; (R10-R12) three session constants. This model was estimated for the seed regions amygdala, ACC and DLPFC.

PPI 2. This GLM tested for differential coupling between brain areas in the planning phase as a function of reward type (high fat vs. low fat). The results are shown in Figure 4E. The model

contained the following regressors: (R1) a psychophysiological interaction regressor between the time series of activity in a seed brain area, extracted as just described, and a contrast between the planning phase trials in which high fat cues were shown and planning phase trials in which low fat cues were shown; (R2) the time series of activity in a seed brain area, extracted as just described; (R3) a contrast between the planning phase trials in which high fat cues were shown and planning phase trials in which low fat cues were shown; (R4-R9) the motion parameters resulting from the realignment pre-processing step as covariates of no interest; (R10-R12) three session constants. This model was estimated for the seed regions amygdala, ACC and DLPFC.

PPI 3. This GLM tested for differential coupling between brain areas in the planning phase as a function of interest rate (high interest vs. low interest). The results are shown in Figure 4E. The model contained the following regressors: (R1) a psychophysiological interaction regressor between the time series of activity in a seed brain area, extracted as just described, and a contrast between the planning phase trials in which high interest cues were shown and planning phase trials in which low interest cues were shown; (R2) the time series of activity in a seed brain area, extracted as just described; (R3) a contrast between the planning phase trials in which high interest cues were shown and planning phase trials in which low interest cues were shown; (R4-R9) the motion parameters resulting from the realignment pre-processing step as covariates of no interest; (R10-R12) three session constants. This model was estimated for the seed regions amygdala, ACC and DLPFC.

PPI 4. This GLM tested for differential coupling between brain areas in the choice phase as a function of current-trial choice (save vs. spend). The results are shown in Figure 5I. The model contained the following regressors: (R1) a psychophysiological interaction regressor between the time series of activity in a seed brain area, extracted as just described, and a contrast between the choice phase trials in which the subject chose to save and choice phase trials in which the subject chose to spend; (R2) the time series of activity in a seed brain area, extracted as just described; (R3) a contrast between the choice phase trials in which the subject chose to save and choice phase trials in which the subject chose to spend; (R4-R9) the motion parameters resulting from the realignment pre-processing step as covariates of no interest; (R10-R12) three session constants. This model was estimated for the seed regions amygdala, ACC, DLPFC, and MPFC.

For all models, the regressors were constructed using the standard deconvolution procedure as implemented in SPM8 [S8]. For each model, we calculated single-subject first-level contrasts for the PPI regressor (R1) that were then entered into a second level analysis by calculating a one-sample t-test across the single subject coefficients. The results are shown in Figure 4E, Figure 5I and Table S5.

Statistical significance testing. For all fMRI analyses, we report effects that survive correction for multiple comparisons across the whole brain using a significance level of $P < 0.05$ (family-wise error) at cluster level, imposed on maps that were displayed at $P < 0.005$ with minimum cluster size $k = 10$ voxels. In addition, we used small volume correction ($P < 0.05$, cluster-level) in the amygdala, for which we had strong *a priori* hypotheses based on previous human fMRI [S9] and animal single-neuron recording [S3, S4] studies. Small volume correction was performed in a sphere of 6 mm radius that was centred on specific amygdala coordinates [18, -6, -22] reported in a previous fMRI study on food reward and decision-making [S9]. (Very similar coordinates for amygdala activation are found across several studies involving food reward or decision-making [S2, S10, S11].)

Region of interest analysis. We produced time courses from region of interest (ROI) analyses according to the following method [S12]. We extracted raw BOLD data from ROI coordinates based on group clusters, which we defined independently for each subject using a leave-one-out procedure. (We re-estimated the second-level analysis 23 times, each time leaving out one subject to define the ROI coordinates for the left-out subject.) Following data extraction we applied a high-pass filter with a cut off period of 128 s. The data were then z-normalized, oversampled by a factor of 10 using sinc-interpolation, and separated into trials to produce a matrix of trials against time. We generated separate matrices for each event of interest (e.g. onset of planning phase or choice phase). We then fitted GLMs to each oversampled time point across trials separately in each subject. The GLMs were designed to test specific hypotheses as described in the text. In addition to the regressors shown in each figure, the GLMs included motion parameters as covariates of no interest. This GLM analysis yielded one regression coefficient for each regressor for every oversampled time point in each subject. We entered individual-subject coefficients into one-sample t-tests (random-effects analysis, $P < 0.05$) and calculated group averages and standard errors for each time point across participants, yielding the across-subject effect size time courses shown in the figures. These mean effect size time courses are shown for the amygdala in Figure 2D and 5B and Figure S4C-F,H and Figure 3B;

the DLPFC time courses are shown in Figure 4C, 5D; the ACC time courses are shown in Figure 5F; the MPFC time courses are shown in Figure 5H.

For the time courses shown in Figure 2F, we performed the following analysis. First, in a ROI analysis (as just described), for each subject we regressed sequence length and sequence value on the oversampled BOLD data for each time point in the planning phase. This yielded regression coefficients for sequence value and sequence length for each time point during the planning phase. We then used these regression coefficients to fit two models to amygdala activity to obtain predicted (i.e. modelled) amygdala activity based on sequence length ('sequence length signal') and sequence value ('sequence value signal'). To relate these two signals to the willingness-to-pay (BDM) bids obtained in the auction-like task, we calculated a sequence length signal and a sequence value signal as just described for all specific saving sequences chosen by each subject (a specific saving sequence for this analysis was defined by sequence length and experimental condition, i.e. the combination of reward type and interest rate). We then regressed the willingness-to-pay (BDM) bids for each specific saving sequence on the corresponding sequence length and sequence value signals, separately for each time point in the planning phase and for each subject. We entered individual-subject coefficients into one-sample t-tests (random-effects analysis, $P < 0.05$) and calculated group averages and standard errors for each time point across participants, yielding across-subject effect size time courses. The resulting mean effect size time courses are shown in Figure 2F.

The region-of-interest analysis in Figure S4C was done to show the effect of sequence length in amygdala when variance related to reward type had been accounted for. We tested for reward effects with a direct indicator variable for fat content across all sequences (1 = high fat; 0 = low fat) and then entered sequence length as second regressor, orthogonalizing sequence length with respect to reward type. The figure thus shows that amygdala planning activity reflects sequence length even if sequence length variation due to reward type is removed. Using the same approach, the analysis in Figure S4D was done to show the effect of sequence length in amygdala when variance related to interest rate had been accounted for. The figure thus shows that amygdala planning activity codes sequence length even if sequence length variation due to interest rate is removed. Together, these control analyses show that our main effect of amygdala sequence length coding in the planning phase is not explained by simple effects of either reward type or interest rate (or related cue responses). Rather, amygdala planning activity seems to reflect the internally planned, forthcoming length of the current sequence.

To test for relationships between specific behavioral and neural effect sizes, we extracted neural effects sizes from individual subject's data using the leave-one-out procedure described above. The resulting effect size scatter plots are shown in Figures 3C, 4D, 4F, 5J, Figures S5D-F. This method was also used to obtain the correlations between PPI effect sizes stated in the main text.

Shared variance and relationship between our main variables. We calculated the shared variance between our main regressors for fMRI data analysis within each subject. The shared variances were as follows: sequence length and sequence value: $R^2 = 0.22$ (± 0.16); sequence length and willingness-to-save ratings: $R^2 = 0.58$ (± 0.13); sequence length and BDM bids (correlations involving BDM bids were calculated for those 22 subjects for whom BDM data were available): $R^2 = 0.36$ (± 0.28); sequence value and willingness-to-save ratings: $R^2 = 0.18$ (± 0.13); sequence value and BDM bids: $R^2 = 0.37$ (± 0.28).

SUPPLEMENTAL REFERENCES

S1. Grabenhorst, F., and Rolls, E.T. (2014). The representation of oral fat texture in the human somatosensory cortex. Human Brain Mapping *35*, 2521-2530.

S2. Grabenhorst, F., Rolls, E.T., Parris, B.A., and d'Souza, A.A. (2010). How the Brain Represents the Reward Value of Fat in the Mouth. Cereb Cortex *20*, 1082-1091.

S3. Grabenhorst, F., Hernádi, I., and Schultz, W. (2012). Prediction of economic choice by primate amygdala neurons. Proc Natl Acad Sci USA *109*, 18950-18955.

S4. Hernadi, I., Grabenhorst, F., and Schultz, W. (2015). Planning activity for internally generated reward goals in monkey amygdala neurons. Nat Neurosci *18*, 461-469.

S5. Becker, G., DeGroot, M., and Marschak, J. (1964). Measuring utility by a single-response sequential method. Behavioral Science *9*, 226-232.

S6. Deichmann, R., Gottfried, J.A., Hutton, C., and Turner, R. (2003). Optimized EPI for fMRI studies of the orbitofrontal cortex. NeuroImage *19*, 430-441.

S7. Friston, K.J., Buechel, C., Fink, G.R., Morris, J., Rolls, E., and Dolan, R.J. (1997). Psychophysiological and Modulatory Interactions in Neuroimaging. NeuroImage *6*, 218-229.

S8. Gitelman, D.R., Penny, W.D., Ashburner, J., and Friston, K.J. (2003). Modeling regional and psychophysiologic interactions in fMRI: the importance of hemodynamic deconvolution. Neuroimage *19*, 200-207.

S9. Grabenhorst, F., Schulte, F.P., Maderwald, S., and Brand, M. (2013). Food labels promote healthy choices by a decision bias in the amygdala. NeuroImage *74*, 152-163.

S10. De Martino, B., Kumaran, D., Seymour, B., and Dolan, R.J. (2006). Frames, Biases, and Rational Decision-Making in the Human Brain. Science *313*, 684-687.

S11. Sharot, T., Riccardi, A.M., Raio, C.M., and Phelps, E.A. (2007). Neural mechanisms mediating optimism bias. Nature *450*, 102-105.

S12. Behrens, T.E., Hunt, L.T., Woolrich, M.W., and Rushworth, M.F. (2008). Associative learning of social value. Nature *456*, 245-249.
